# Biological Evaluations and Computer-Aided Approaches of Janus Kinases 2 and 3 Inhibitors for Cancer Treatment: A Review

**DOI:** 10.3390/pharmaceutics16091165

**Published:** 2024-09-04

**Authors:** Lenci K. Vázquez-Jiménez, Gildardo Rivera, Alfredo Juárez-Saldivar, Jessica L. Ortega-Balleza, Eyra Ortiz-Pérez, Elena Jaime-Sánchez, Alma Paz-González, Edgar E. Lara-Ramírez

**Affiliations:** 1 Laboratorio de Biotecnología Farmacéutica, Centro de Biotecnología Genómica, Instituto Politécnico Nacional, Reynosa 88710, Mexico; giriveras@ipn.mx (G.R.); ajuarezs1500@gmail.com (A.J.-S.); jessica_ortega7@hotmail.com (J.L.O.-B.); eortizp@ipn.mx (E.O.-P.); nena_smile@live.com (E.J.-S.); apazg@ipn.mx (A.P.-G.); 2 Consejo Nacional de Humanidades, Ciencias y Tecnologías (CONAHCYT), Mexico City 03940, Mexico

**Keywords:** anticancer, compounds, JAK2, JAK3

## Abstract

Cancer remains one of the leading diseases of mortality worldwide. Janus kinases 2/3 (JAK2/3) have been considered a drug target for the development of drugs to treat different types of cancer. JAK2/3 play a critical role in innate immunity, inflammation, and hematopoiesis by mediating the signaling of numerous cytokines, growth factors, and interferons. The current focus is to develop new selective inhibitors for each JAK type. In this review, the current strategies of computer-aided studies, and biological evaluations against JAK2/3 are addressed. We found that the new synthesized JAK2/3 inhibitors are prone to containing heterocyclic aromatic rings such as pyrimidine, pyridine, and pyrazolo [3,4-*d*]pyrimidine. Moreover, inhibitors of natural origin derived from plant extracts and insects have shown suitable inhibitory capacities. Computer-assisted studies have shown the important features of inhibitors for JAK2/3 binding. Biological evaluations showed that the inhibition of the JAK receptor affects its related signaling pathway. Although the reviewed compounds showed good inhibitory capacity in vitro and in vivo, more in-depth studies are needed to advance toward full approval of cancer treatments in humans.

## 1. Introduction

Cancer is one of the most serious diseases that cause death worldwide, being an important and serious medical problem [1]. Cancer is characterized by an unprogrammed and uncontrolled cell multiplication process [2] in which the failure of various coding genes for antiapoptotic, growth factor receptors, tumor suppressors, and transcription factor proteins are implicated. These dysregulated molecules are one of the main targets for cancer prevention, diagnosis, and treatment [1].

The Janus kinase (JAK) proteins and the signal transducer and activator of transcription (STAT) are important signaling molecules involved in altered tissue gene expression, making them targets of great interest for the drug discovery efforts to treat cancer. The JAK non-receptor tyrosine kinases participate in the control and regulation of tissue repair process and in immune and hematopoietic signaling pathways [3,4,5]. The JAK family includes JAK1–3 and TYK2 (tyrosine kinase 2); structurally, these proteins are characterized by the exclusive tandem kinase domain, the regulatory pseudokinase domain (JH2), and the tyrosine kinase domain (JH1). The JAK FERM-SH2 domains located at the N-terminus bind to their cytokine receptors, which lack catalytic activity. Thus, the formation of a cytokine receptor–JAK signaling complex initiates the associated intracellular signaling cascade [6,7].

Particularly, JAK2/3 are suitable targets for the treatment of hematologic tumors and myeloproliferative neoplasms [8,9]; however, the discovery of JAK2/3 inhibitors remains a challenge. Currently, most JAK2/3 inhibitors are still in clinical studies [10,11], for example, ruxolitinib, pacritinib, and AZD1480 (Table 1) [12,13]. Among them, ruxolitinib was the first JAK2 inhibitor approved for its use in humans to treat primary myelofibrosis [14]. These JAK inhibitors have shown good efficacy for some diseases, but their adverse effects have led to clinical safety concerns (Table 1) [8,12,13,14,15].

In the case of JAK3, some inhibitors have also been described, for example, Ritlecitinib, acrylamide-based inhibitor PF-06651600 (Table 2), has been investigated in various phase II and III clinical trials [16,17]. Tofacitinib (Table 2), first described as selective for JAK3 [18], was shown to be a pan-JAK inhibitor [19,20]. Although there have been clear benefits obtained in patients treated with pan-JAK inhibitors, some adverse events have also been observed (Table 2) [21].

## 2. The JAK2 Target

JAK2 is a type of intracellular protein with tyrosine kinase activity present in different cells and tissues [22]. JAK2 has essential functions for red blood cells and platelet production in the bone marrow [23,24]. JAK2 is related to and communicates with hormone-like cytokines through interferons and receptors containing the glycoprotein 130 [25].

**Figure 1:**
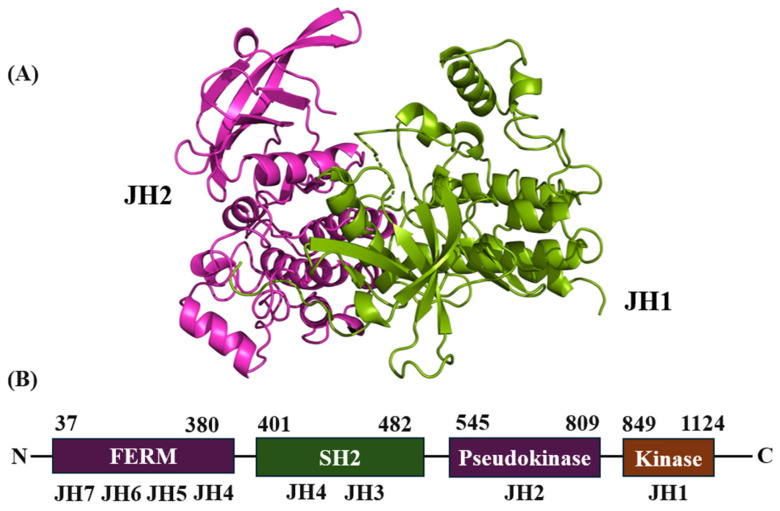
Structure of the JAK2 kinase domain (PDB ID: 8BM2, JH1; 7F7W, JH2) (**A**) and domain structure of JAK2 (**B**). Magenta color indicates the JH2 domain, and green color represents the JH1 of JAK2. The 3D structure image was generated with PyMOL software v. 3.0.3 (https://www.pymol.org/).

Structurally, JAK2 had a hinge that connects the β-sheet N-lobe and C-lobe. The N-lobe is responsible for the binding and release of ATP/ADP [26,27]. Seven JH domains have been described based on their functionality. In the C-terminal direction, the catalytic JH1 domain is located; in the same direction, the JH2 domain (regulatory pseudokinase domain) essential for several regulatory functions is located, followed by JH3–JH5; the latter promotes binding to cytokine receptors. The JH6 and JH7 domains located at the N-terminal, close to the domain similar to the homology Src-2 (SH2), also mediate the interactions with the subunits of cytokine receptors (Figure 1) [28]. JAK2 is a central protein that mediates extracellular signals given by interleukin receptors and tyrosine kinases to pass the signals to the oncogenic transcription factor called signal transducer and activator of transcription 3 (STAT3) [29,30]. This is achieved by phosphorylating the amino acid Tyr705 of STAT3 that produces the homodimerization of p-STAT3 through the interaction of its phosphorylated sites, inducing its nuclear translocation and transcriptional activity [30,31,32].

Research on the JH2 domain showed it is an important molecular regulatory site for the structure of the JAK protein [33,34]. For example, JH2 are related to hematologic diseases [28]. In particular, the V617F JAK2 mutation, which is found in most patients with polycythemia vera, essential thrombocythemia, and idiopathic myelofibrosis, makes this kinase a potential therapeutic target to treat patients suffering from these types of cancer [35,36,37,38,39]. In fact, JAK2 inhibition promotes the termination of cell progression and ultimately apoptosis in cancer cell line studies [40,41]. JAK2 inhibitors are classified into two main classes: type 1 that binds to the ATP-binding site in the active conformation and type 2 inhibitors that bind to the inactive conformation [25,42]. Furthermore, most JAK2 inhibitors form hydrogen bonds with Leu932 and Asp994, which are important interactions for the inhibition of JAK2 [43].

## 3. The JAK3 Target

**Figure 2:**
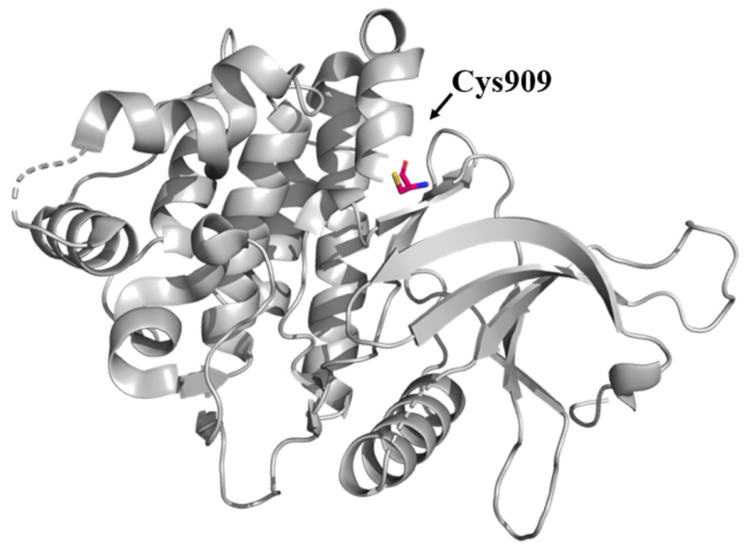
Cys909 residue (sticks) in the JAK3 structure (PDB ID: 5TTV).

JAK3 is expressed mainly in hematopoietic cells such as lymphoid cells and participates in the common γ chain (γc) to release the cytokines IL-2, IL-4, IL-7, IL-9, IL-15, and IL-21 for the development of B, T, and natural killer cells [44,45]. JAK3 also influences the antiapoptotic PI3K-AKT signaling pathway and its related downstream targets, some of which have been described as oncogenic proteins [45]. JAK3 dysfunction (R925S and Q988P) has been associated with acute lymphoblastic leukemia and the pathogenesis of lymphoid-derived diseases [5,46]. For example, in several hematological cancer cell lines, such as anaplastic large cell lymphoma, Burkitt lymphoma, mantle cell lymphoma, and enteropathy-associated T-cell lymphoma, JAK3/STAT signaling is aberrantly activated [47,48,49,50]. In the search for JAK3 inhibitors, cysteine αD-1 (Cys909) has attracted considerable interest (Figure 2). It is an important feature that distinguishes JAK3, because cysteine replaced a serine residue at the equivalent position in the other JAK-type proteins [51], this being an option in the search for selective covalent JAK3 inhibitors.

## 4. JAK2 Inhibitors

**Figure 3:**
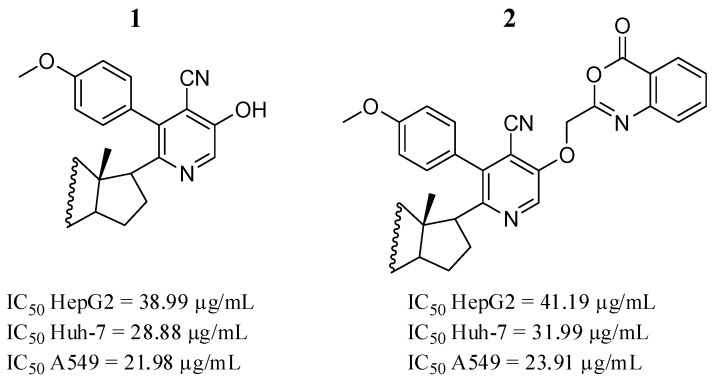
Chemical structures of heterosteroid derivatives **1** and **2** described by Mohamed et al. [52]. In the figure, the numbers in bold refer to the compounds described in the text, indicating their inhibitory values in distinct cancer cell lines. Figures were drawn and labeled with ChemDraw v. 19.0.

One of the strategies to search for new cancer treatments has been the synthesis of hybrid anticancer agents. Mohamed et al. [52] investigated the anticancer activity of synthetically hybrid heterosteroid derivatives against human liver cell lines derived from hepatocellular carcinoma (HepG2 and Huh-7), as well as in non-small cell lung cancer cell lines (A549). Compounds **1** and **2** (Figure 3) had the lowest IC_50_ and the highest cytotoxic effects against all cell lines tested. Furthermore, they performed a molecular docking analysis to predict the activity of the tested ones against the JAK2 protein, where compounds **1** and **2** showed docking scores of −11.3 and −12.0 Kcal/mol. Both compounds interacted with JAK2 mainly through hydrophobic interactions with amino acids Leu932, Leu983, Leu982, Leu997, Leu855, Val911, Val863, and Phe860. Compound **1** forms two hydrogen bonds with Leu932, and compound **2** showed an interaction with Lys882 through a hydrogen bond. SAR analysis revealed that the remarkable effects of compounds **1** (nicotinonitrile derivative) and **2** (oxazinone derivative) may be due to the steroid moiety.

**Figure 4:**
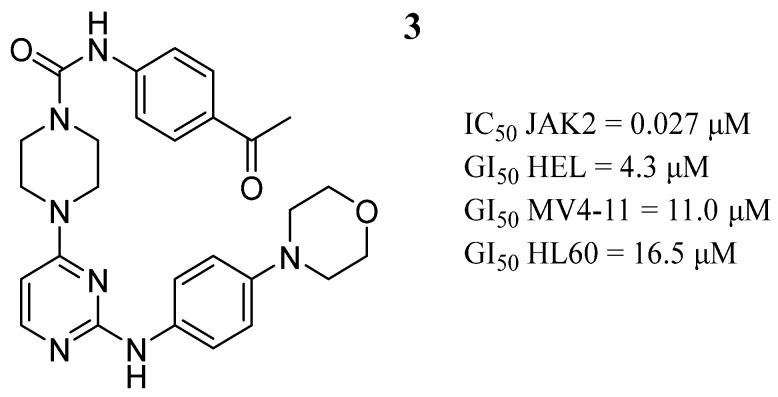
Chemical structure of the lead 4-piperazinyl-2-aminopyrimidine derivative described by Li et al. [53]. In the figure, the number in bold refers to the compound described in the text, indicating its inhibitory values in JAK2 and distinct cancer cell lines. The figure was drawn and labeled with ChemDraw v. 19.0.

Li et al. [53] linked the momelotinib and tandutinib pharmacophores to obtain a series of 4-piperazinyl-2-aminopyrimidine derivatives. Compound **3** showed in vitro inhibitory activity against JAK2 and the HEL, MV4-11, and HL60 cancer cell lines (Figure 4). In the cellular context, compound **3** in HL cells induced apoptosis in a dose-dependent manner and produced cell cycle arrest at the G1/S phase. Computer-aided studies showed that compound **3** binds to the ATP-binding site; in the hinge region, the 2-aminopyrimidine moiety formed two hydrogen bonds with the Leu932 residue. Meanwhile, the pyrimidinyl group formed a network-like alkyl-π interaction with the adjacent amino acids Ala880, Leu855, Val863, and Leu983. The morpholine moiety formed a hydrogen bond with Lys943 and was exposed to the solvent area, and another hydrogen bond was formed between the NH group of N-phenylpiperazine-1-carboxamide and Leu855 amino acid.

**Figure 5:**
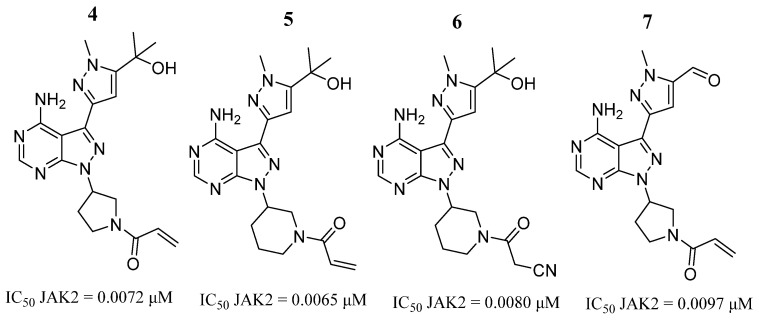
Chemical structure of 1H-pyrazolo[3,4-d]pyrimidin-4-amino derivatives synthesized by Yin et al. [39]. In the figure, the numbers in bold refer to the compounds described in the text, indicating their inhibitory values in JAK2. Figures were drawn and labeled with ChemDraw v. 19.0.

Yin et al. [39], through the introduction of a 3,5-disubstituted-1*H*-pyrazole fragment at the C-3 position of the pyrazole structure, synthesized a series of 1*H*-pyrazolo [3,4-*d*]pyrimidin-4-amino derivatives. Compounds **4**, **5**, **6**, and **7** showed activity against the JAK2 enzyme, with IC_50_ values of 6.5 to 9.7 nM (Figure 5). Compound **6** exhibited the most potent antiproliferative activities against K562 cells, with an IC_50_ value of 8.67 μM. Compound **5** showed higher potency against the HEL cell line (IC_50_ = 6.46 µM) (Figure 5). SAR analysis showed that a substitution at the *meta* position is beneficial for bioactivity, where acrylamide (**5**) and cyanoacetamide (**6**) resulted in similar potency and selectivity against JAK2. Finally, they performed a molecular docking analysis of compound **5**, where the pyrazolopyrimidine ring binds to the hinge region through two hydrogen bonds with Leu932 and two π-π interactions with Tyr931. The piperidine-3-yl group fits into the active site delimited by the amino acids Leu855, Gly856, Lys857, Val863, Met865, and Phe995. Meanwhile, the acrylamide group is attached to the kinase through a hydrogen bond with Lys857. At the same time, two additional hydrogen bonds were observed between 4-aminopyrimidine and the pyrazole of compound **5** and residue Tyr931. The hydrogen bonding network generated by 2-hydroxypropane with Ser936 and Asp939 through a water molecule, near the ATP-binding site, was suggested to be important for the observed inhibitory activity and selectivity.

**Figure 6:**
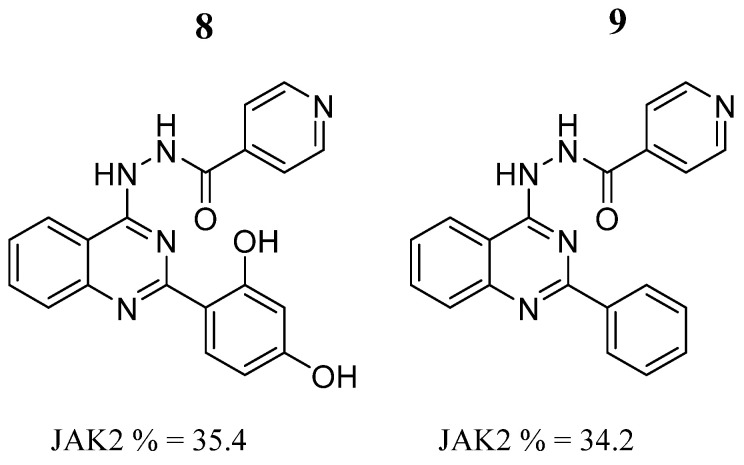
Chemical structures of 2,4-disubstituted quinazoline derivatives synthesized by Jyothi-Buggana et al. [54]. In the figure, the numbers in bold refer to the compounds described in the text, indicating their inhibitory values in JAK2. Figures were drawn and labeled with ChemDraw v. 19.0.

Jyothi-Buggana et al. [54] synthesized a series of 2,4-disubstituted quinazolines and evaluated their cytotoxicity against human breast cancer (MDA-MB-231) and ovarian cancer (SK-O-V3) cell lines. Compound **8** (Figure 6) showed better cytotoxic activity against breast cancer cell lines (IC_50_ = 10.1 μM). The in vitro JAK2 inhibition assay reported that compounds **8** and **9** had about 35% inhibition of the JAK2 enzyme (Figure 6). SAR studies indicated that a hydrophobic aromatic group linked with hydroxyl or nitro hydrophilic substituents could increase the cytotoxic effects. Molecular docking studies in the kinase domain showed that compound **8** had the highest docking score, being −8.63 Kcal/mol, and compound **9**, −7.70 Kcal/mol. Both showed hydrogen bond interactions with Leu932. The two compounds had common chemical groups characterized by a phenyl ring and the hydrazine nitrogen of the isonicotinohydrazide, attached to the quinazoline moiety, that was involved in hydrogen bonding with the protein. The carbonyl groups attached to quinazoline also have hydrophilic interactions with the protein. They also identified that Arg938 had a hydrophobic interaction with the ligands. Furthermore, chloroquinazoline chlorine tends to have ionic interactions with Asp994. During molecular dynamics simulations of the JAK2-**8** and JAK2-**9** complexes, they observed that the RMSD patterns were different in a simulation of 20 ns. The RMSD of Cα and the side chain amino acids suggested that compound **8** remained stable with an overall deviation < 1 Å; in contrast, compound **9** showed more fluctuations with an overall deviation > 2 Å, suggesting that a longer simulation time can elucidate the binding pattern and fate of ligand **9**. During the simulation period, Arg980 (97%), Lys857 (87%), and Leu932 (59%) are the amino acids that interact the most with compound **8**, while residues Leu855 (74%) and Ser936 (72%/71%) interact the most with compound **9** [54].

**Figure 7:**
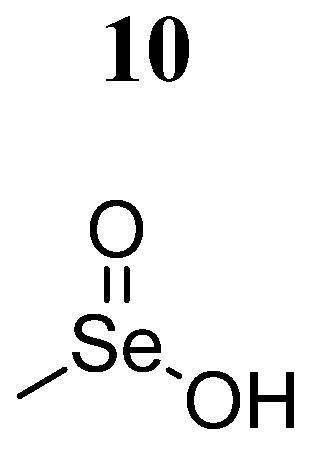
Chemical structure of methylseleninic acid (**10**). In the figure, the number in bold refers to the compound described in the text. The figure was drawn and labeled with ChemDraw v. 19.0.

Previous studies have shown that methylseleninic acid (MSA) (compound **10**) (Figure 7) exerts specific cytotoxic activity in different cancer cells [55,56]. Zhang et al. [57] evaluated compound **10** in 4T1 cells and mouse tumor models. It was demonstrated that MSA inhibits cell viability in a concentration- (5, 10, and 20 μmol/L) and time (6, 12, and 24 h)-dependent manner. MSA activated caspase-3, poly ADP ribose polymerase 1 (PARP1), and BCL2. X, generating apoptotic effects. Furthermore, the use of the chemical JAK2 inhibitor AG490 as a positive control in comparison with compound **10** demonstrated anticancer activity by inhibiting the JAK2/STAT3 pathway, evidenced by the decrease in JAK2 and STAT3 phosphorylation in 4T1 cells. In vivo studies through morphological and TdT-mediated dUTP end labeling analysis showed that compound **10** (1.5 mg/kg/wt) inhibited tumor growth according to the clinical anticancer drug cyclophosphamide.

**Figure 8:**
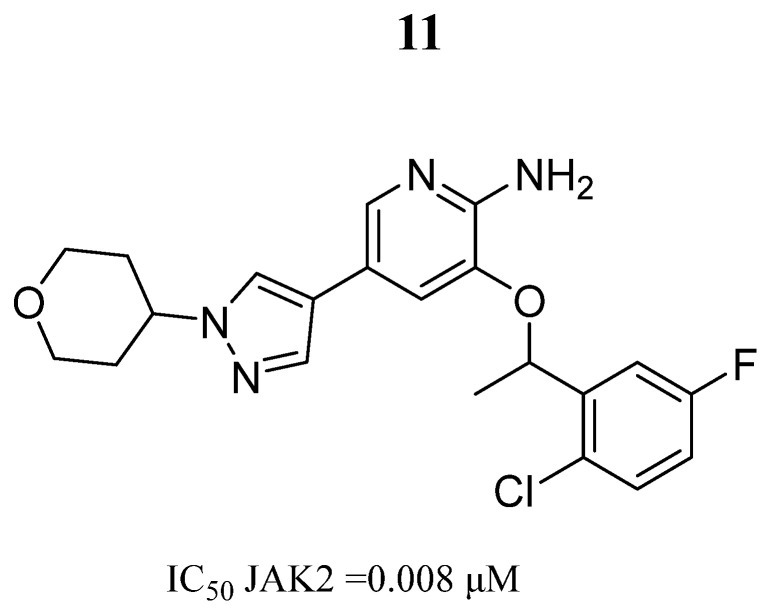
Chemical structure of the lead 2-aminopyridine derivative evaluated by Ma et al. [58]. In the figure, the number in bold refers to the compound described in the text, indicating its inhibitory value in JAK2. The figure was drawn and labeled with ChemDraw v. 19.0.

In 2020, Ma et al. [58] carried out the design and synthesis of a series of 2-aminopyridine derivatives and their biological evaluation. Compound **11**, a two-optical isomer resolved by enantiomeric resolution, showed notable potency against JAK2 (Figure 8) and binds stably to the JAK2 ATP-binding pocket. After evaluating the enzymatic activity, they found that the R enantiomer was the most potent and selective compound, exhibiting an IC_50_ of 3.0 nM against JAK2 and a selectivity of 85 and 76 times over JAK1 and JAK3, respectively. The SAR analysis indicated that the oxygen atoms in the rings were related to inhibitory effects against JAK2.

**Figure 9:**
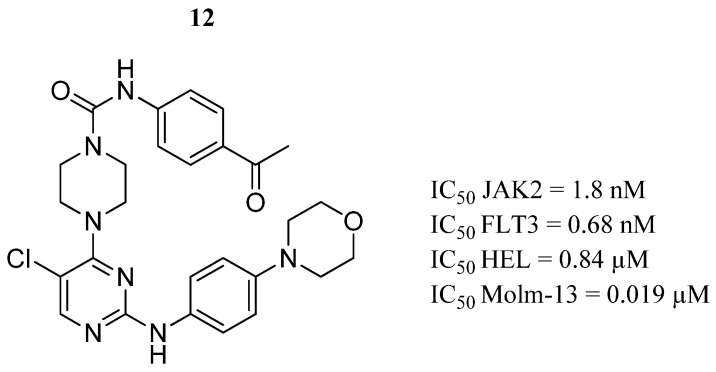
Chemical structure of the 2-aminopyrimidine derivative analyzed by Li et al. [59]. In the figure, the number in bold refers to the compound described in the text, indicating its inhibitory values in JAK2 and cancer cell lines. The figure was drawn and labeled with ChemDraw v. 19.0.

In the same year, Li et al. [59] carried out rational drug design based on the JAK2 structure, synthesized, and evaluated a series of 2-aminopyrimidine derivatives. The derivative compound **12** demonstrated the highest inhibitory activity against JAK2 and tyrosine kinase 3 (FLT3) (Figure 9). Compound **12** showed biological effects in the HEL and Molm-13 cancer cell lines but weak cytotoxicity in the K562 and PC-3 cell lines (IC_50_ = 4.3 and >20 μM, respectively), suggesting target specificity. Furthermore, in the Molm-13 cell line, compound **12** induced cell cycle arrest in the G1/S phase and apoptotic effects in a dose-dependent manner. In the metabolism assay in rat liver microsomes, compound **12** had a half-life time of 31 min, indicating moderate stability. Computer simulations showed compound **12** bound to the JAK2 ATP-binding pocket. 2-aminopyrimidine formed a hydrogen bond with the Leu932 amino acid located in the hinge region. Meanwhile, the 6-chloropyrimidine group formed hydrophobic interactions with Val863, Ala880, Val911, Met929, and Leu983 residues in a network-like appearance. The morpholine moiety exposed in the cartridge area formed a hydrogen bond with Lys943, and along with the carbonyl tail group, two other hydrogen bonds with Asp976 and Arg980 amino acids were formed.

**Figure 10:**
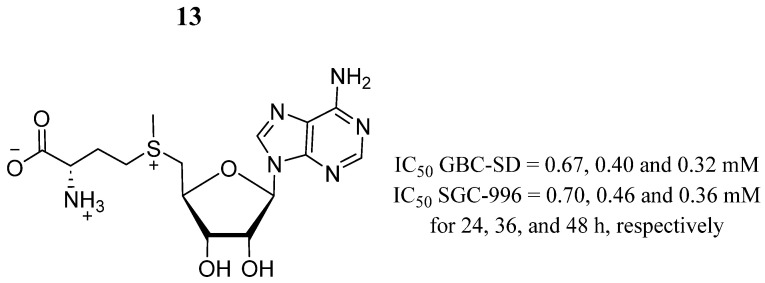
Chemical structure of S-adenosylmethionine (**13**). In the figure, the number in bold refers to the compound described in the text, indicating its inhibitory values in distinct cancer cell lines over time. The figure was drawn and labeled with ChemDraw v. 19.0.

S-adenosylmethionine (SAM) (compound **13**) (Figure 10) is a natural metabolite found ubiquitously in mammalian cells [60] and is a reagent for the development of drug cancer treatments [61,62,63,64]. Liu et al. [65] determined that compound **13** inhibited growth and proliferation in GBC-SD and SGC-996 gallbladder cancer cells in a dose- and time-dependent manner after treatment for 24, 36, and 48 h (Figure 10). This compound also caused apoptosis and cell cycle arrest in the G0/G1 phases in both types of cell lines. Furthermore, the expression levels of phosphorylated JAK2 (p-JAK2) were significantly reduced. Further JAK2/STAT3 pathway inhibitory tests demonstrated enhanced antiapoptotic effects, suggesting the key role of SAM for modulating this important signaling pathway. Finally, in vivo studies demonstrated that SAM decreased tumor size, and the immunohistochemical staining revealed the downregulation of p-JAK2 in the tumor tissues.

**Figure 11:**
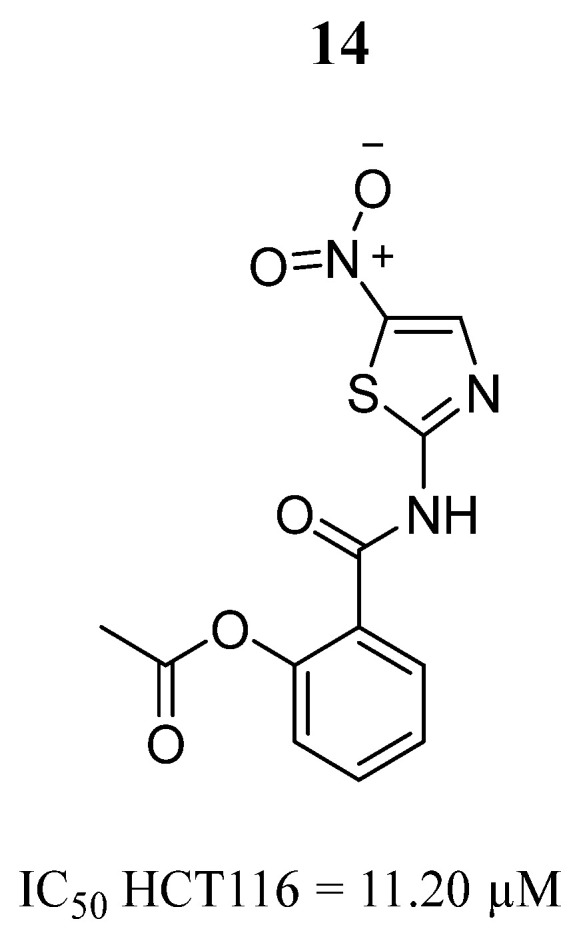
Chemical structure of 2-((5-nitrothiazol-2-yl)carbamoyl)phenyl acetate (**14**). In the figure, the number in bold refers to the compound described in the text, indicating its inhibitory values in a cancer cell line. The figure was drawn and labeled with ChemDraw v. 19.0.

Another compound that has been evaluated as an anticancer agent against cancer of the ovarian, colon, and glioblastoma is nitazoxanide (**14**) (Figure 11), an antiparasitic drug approved by the Food and Drug Administration (FDA) [66,67,68]. Tantawy et al. [69] examined the in vitro antiproliferative and apoptotic effects of compound **14** on the human colorectal cancer cell line HCT116 alone or in combination with compound 5-fluorouracil (5-FU), the standard drug for the treatment of human colorectal cancer. The in vitro results showed that compound **14** had better inhibitory effects than 5-FU (Figure 11) and produced downregulation of the signaling molecules IL6/JAK2/STAT3. The molecular docking prediction indicated that compound **14** had better binding affinity than 5-FU (−7.8 and −5.0 Kcal/mol, respectively). Compound **14** formed a hydrophobic interaction with the amino acids Leu932, Leu983, Val863, Val911, and Phe863 and a hydrogen bond with the Arg980 amino acid. Based on these results, these authors considered that compound **14** could be a promising anticancer agent against colorectal cancer.

**Figure 12:**
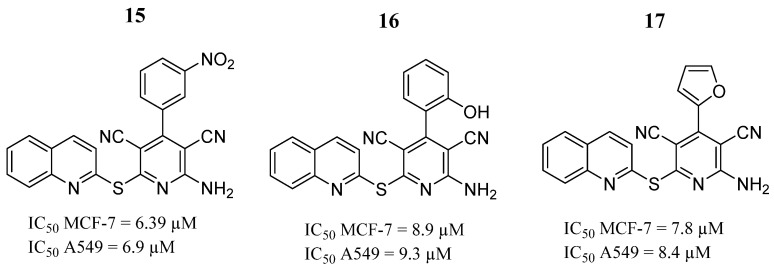
Chemical structure of the 2-amino-4-aryl-6-(quinolin-2-ylthio)pyridine-3,5-dicarbonitrile derivatives synthesized by Nafie et al. [70]. In the figure, the numbers in bold refers to the compound described in the text, indicating their inhibitory values in distinct cancer cell lines. Figures were drawn and labeled with ChemDraw v. 19.0.

Nafie et al. [70], by a single-phase reaction, synthesized various 2-amino-4-aryl-6-(quinolin-2-ylthio)pyridine-3,5-dicarbonitrile derivatives. In the presence of sodium hydroxide in absolute ethanol, these derivatives contain quinoline-2-thione, aromatic aldehydes, and malononitrile moieties. Compounds **15**, **16**, and **17** showed the best anticancer activities against MCF-7 and A549 cells. Furthermore, the JAK2 and STAT3 genes were negatively regulated in MCF-7 cells treated with compound **15** (Figure 12). The results of the molecular docking analysis indicated that compound **15** formed hydrogen bonds with the key amino acid Leu932. Furthermore, compound **15** formed lipophilic interactions with the nonpolar amino acid residues Leu983, Leu855, Val863, Pro933, Met929, Ala880, and Leu932 within the JAK2 pocket. Hence, this finding made the authors suggest the mode of action for anti-breast cancer activity.

**Figure 13:**
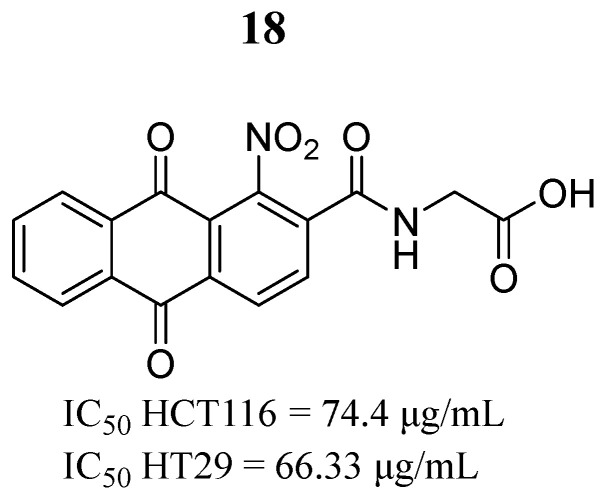
Chemical structure of the compound 1-nitro-2-acetylanthraquinone glycine (**18**). In the figure, the number in bold refers to the compound described in the text, indicating its inhibitory values in distinct cancer cell lines. The figure was drawn and labeled with ChemDraw v. 19.0.

In the same year, Li et al. [10] investigated the effect of the compound 1-nitro-2-acetylanthraquinone glycine (compound **18**) (Figure 13), an anthraquinone previously synthesized [71]. They found that compound **18** had good inhibitory activity in HCT116 and HT29 colon cancer cells (Figure 13), blocking the cell cycle in the G2/M phase by downregulation of cyclin B1 and CDK1 expression. Molecular docking studies showed that compound **18** had a binding score of score of 5.3796 and interacted with Lys882 and Asn859 through three hydrogen bonds in the JAK2 active site. They also showed that compound **18** positively regulates PIAS-3 expression and negatively regulates STAT3 phosphorylation.

**Figure 14:**
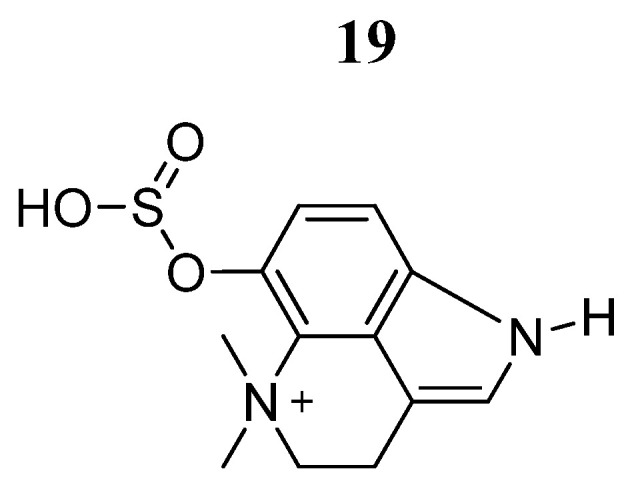
Chemical structure of bufothionine (**19**). In the figure, the number in bold refers to the compound described in the text. The figure was drawn and labeled with ChemDraw v. 19.0.

In 2015, Xie et al. [72] found that buphothionine (**19**) (Figure 14) induced mitochondrial-mediated apoptosis. In 2021, Kong et al. [73] evaluated the effects of compound **19** on the autophagy process in SMMC7721 liver carcinoma cells. Compound **19** showed anticancer activities by promoting apoptosis at 100 μM. In vivo, compound **19** exerted anti-inflammatory activity and alleviated cancer symptoms of H22 tumor-bearing mice. Furthermore, bufothionine reduced the serum concentration of IL-6; suppressed the expressions of STAT3 phosphorylated in Tyr705 and Ser727 amino acids and JAK2 in tumor tissues; and also increased the expression of Atg5, Atg7, and LC3II in SMMC7721 cells and H22 tumors. These results suggested that compound **19** could induce autophagy in hepatocellular carcinoma by inhibiting the JAK2/STAT3 pathway.

**Figure 15:**
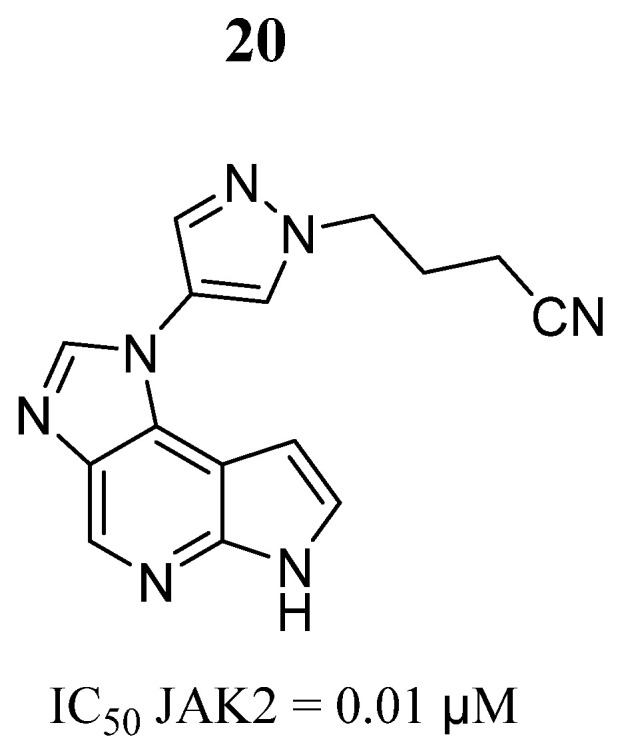
Chemical structure of the lead imidazopyrrolopyridine derivative evaluated by Xu et al. [74]. In the figure, the number in bold refers to the compound described in the text, indicating its inhibitory values in JAK2. The figure was drawn and labeled with ChemDraw v. 19.0.

On the other hand, Xu et al. [74] described the design, synthesis, and molecular docking analysis of a series of imidazopyrrolopyridine derivatives. Compound **20** was a potent inhibitor of JAK2 (Figure 15) and selective against JAK1 (x19), JAK2 (x > 30), and JAK3 (x > 30). Compound **20** inhibited JAK2 signaling (IC_50_ = 0.26 μM) instead of JAK1 signaling (IC_50_ = 10.69 μM). Using a dose of 20 mg/kg, compound **20,** in mice treated with GM-CSF, reduced the phosphorylated STAT3 and STAT5 levels comparable to those of the control animals. Furthermore, compound **20** showed adequate bioavailability (F = 38%), half-life time (T1/2 = 1.9 h), and metabolic stability. The docking simulations showed that the −NH and =N groups of azaindole interacted with Glu930 and Leu932 amino acids through hydrogen bonds; in addition, the cyano group formed hydrogen bonds with Lys882, and these ligand–protein interactions were suggested as important for the inhibition of JAK2.

**Figure 16:**
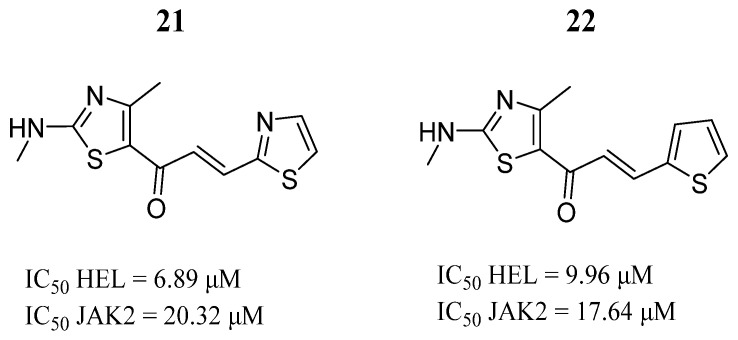
Chemical structures of thiazole aromatic alkylamino analogs evaluated by Sanachai et al. [75]. In the figure, the numbers in bold refer to the compounds described in the text, indicating their inhibitory values in JAK2 and cancer cell lines. Figures were drawn and labeled with ChemDraw v. 19.0.

Sanachai et al. [75] evaluated the anticancer effects of thiazole-based alkylamino aromatic analogs of chalcone in the TF1 and HEL cancer cell lines. Compounds **21** and **22** showed anticancer effects in the HEL cell line but not in TF1 cells (Figure 16). Both compounds inhibited JAK2 in enzyme inhibition assays (Figure 16). The molecular docking showed that these compounds bind to the Lys857, Leu932, and Glu930 amino acids in the hinge region through hydrogen bonds and hydrophobically interact with Leu983 at the catalytic site of JAK2. Hence, those thiazole derivatives could be promising drugs for cancer therapy targeting the JAK2.

**Figure 17:**
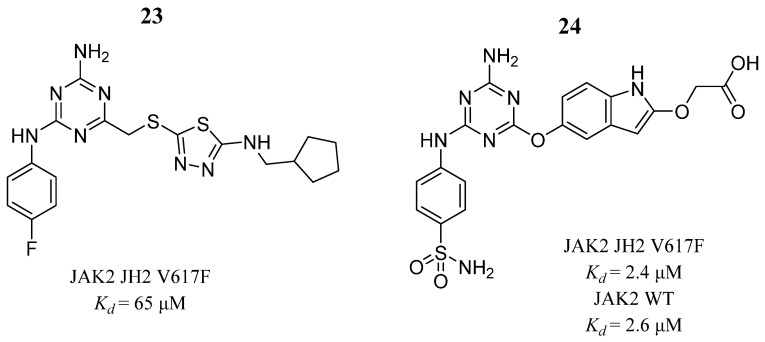
Chemical structure of compounds **23** and **24** analyzed by Newton et al. [76]. In the figure, the numbers in bold refer to the compounds described in the text, indicating their inhibitory values in JAK2. Figures were drawn and labeled with ChemDraw v. 19.0.

In the search for a new JAK2 inhibitor, Newton et al. [76] performed a virtual screening identifying nine compounds that had binding affinities of 40 to 300 μM in a fluorogenic assay. Then, they acquired ten analogs, where aminoanilinyltriazine (**23**) (Figure 17) was particularly notable, because it showed a dissociation constant (*K_d_*) of 65 μM with the V617F JAK2 JH2 mutant. The crystal structure of compound **23** in complex with wild-type JAK2 JH2 showed four hydrogen bonds with Gln626, Glu627, and Val629. Therefore, further synthesis of 19 analogs identified another potent JAK2 inhibitor, compound **24** (Figure 17) with *K_d_* values of 2–3 μM for the wild-type protein and the mutant V617F JAK2 JH2. Molecular docking analysis showed interactions with Gln626, Glu628, Val629, Asn678, Asn673, Thr555, and Arg715.

**Figure 18:**
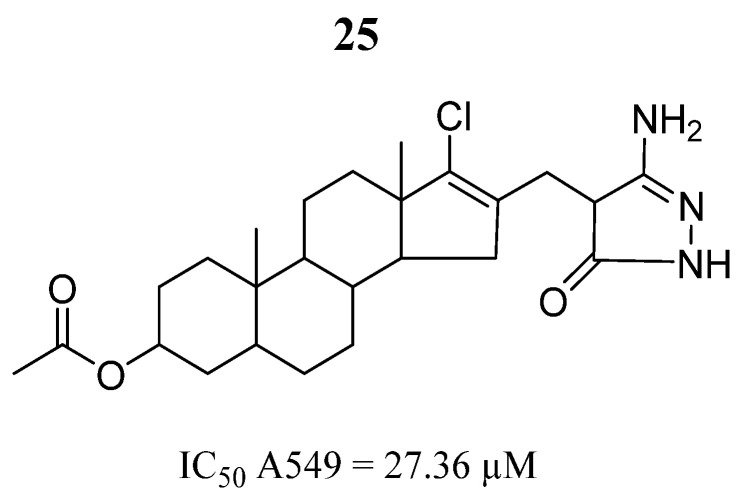
Chemical structure of the 3β-acetoxy-5α-androstane derivative evaluated by Tantawy et al. [77]. In the figure, the number in bold refers to the compound described in the text, indicating its inhibitory values in a cancer cell line. The figure was drawn and labeled with ChemDraw v. 19.0.

Tantawy et al. [77] investigated the anticancer activity of 3β-acetoxy-5α-androstane derivatives on the A549 cancer lung cell line using the MTT assay. Compound **25** had cytotoxic effects (Figure 18) showing cell death by apoptosis in 24.62% and cell cycle arrest in the pre-phases G1 and G2/M. To propose a mode of action, they performed a molecular docking analysis to investigate binding interactions with the JAK2 protein. Compound **25** had a predicted binding energy of −14.16 Kcal/mol, interacting Leu932 amino acid through three hydrogen bonds of a length of 1.73, 1.78, and 2.33 Å. Furthermore, it formed lipophilic interactions with the Leu155, Val24, Ile140, Leu90, and Leu141 amino acids. This was the mode of action suggested for such compounds.

**Figure 19:**
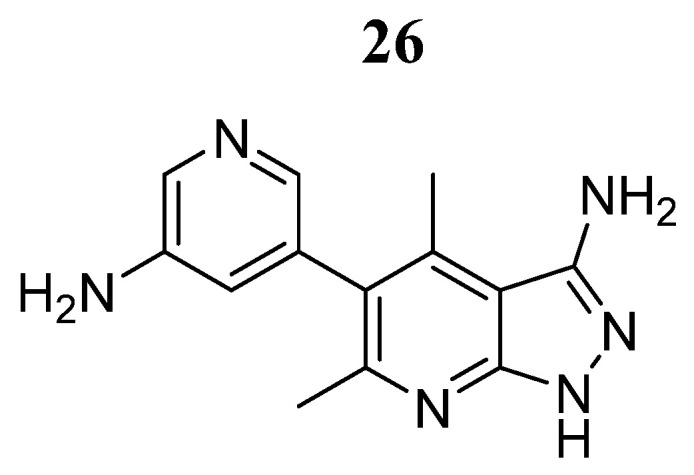
Chemical structure of compound **26** identified by in silico methods as a potential JAK2 inhibitor by Singh et al. [78]. In the figure, the number in bold refers to the compound described in the text. The figure was drawn and labeled with ChemDraw v. 19.0.

Singh et al. [78] conducted an in silico study to identify new potential JAK2 inhibitors from ZINC databases using high-throughput virtual screening techniques. Compound **26** (Figure 19) showed the best docking score (−10.11 Kcal/mol) and a free energy of MM/GBSA (−46.07 Kcal/mol) comparable to that of the known inhibitor ruxolitinib (−9.70 and −46.07 Kcal/mol, respectively). Compound **26** presented hydrogen bond interactions with Glu930 and Leu932 and hydrophobic interactions with the amino acids Leu855, Val863, and Leu983. Furthermore, the molecular dynamics simulations showed a RMSD fluctuation of 1.74 and 0.40 Å, indicating the stability of compound **26** in the binding to JAK2. The average H-bond formation was 3.69 with the amino acids predicted through molecular docking, showing the agreement of the two computational methods.

**Figure 20:**
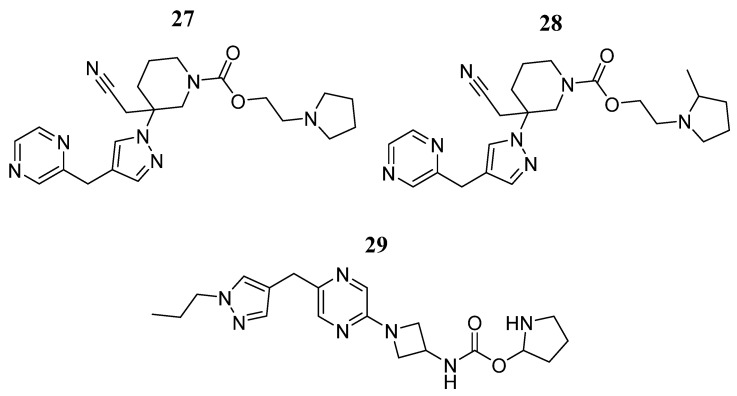
Chemical structure of compounds **27**, **28**, and **29** identified by computational techniques as potential JAK2 inhibitors by He et al. [79]. In the figure, the numbers in bold refer to the compounds described in the text. Figures were drawn and labeled with ChemDraw v. 19.0.

Recent studies have shown that the combination of JAK2 and smoothed receptor (SMO) inhibitors can be used to affect the growth and metastasis of triple-negative breast cancer and drug-resistant HER2-positive breast cancer cells [30]. Therefore, He et al. [79] used deep reinforcement learning to produce a new library of compounds with the potential to inhibit both JAK2 and SMO. This library was screened by molecular docking and the three best compounds ranked on the basis of the docking score were further assessed by molecular dynamics simulation analysis using the MMGBSA method. The top three compounds were compounds **27**, **28**, and **29** (Figure 20), which showed docking scores of −9.43, −9.73, and −9.77, respectively. All three compounds interacted with Leu932 and Asp994 of the JAK2 protein and with Asn219, Tyr394, and Arg400 in the SMO protein. In molecular dynamics, all RMSDs were within 0.2 nm, indicating that the compounds bind steadily at the active site of the two proteins. The MMPBSA results showed that the binding free energy of compounds **27**, **28**, and **29** to JAK2 was −267.47, −218.32, and −255.49 Kcal/mol, respectively, and that of SMO was −158.39, −232.61, and −251.46 Kcal/mol. They also observed that the three compounds formed hydrogen bonds, in agreement with the molecular docking results. In general, the computer-aided studies showed that these compounds could be dual-target anticancer agents.

**Figure 21:**
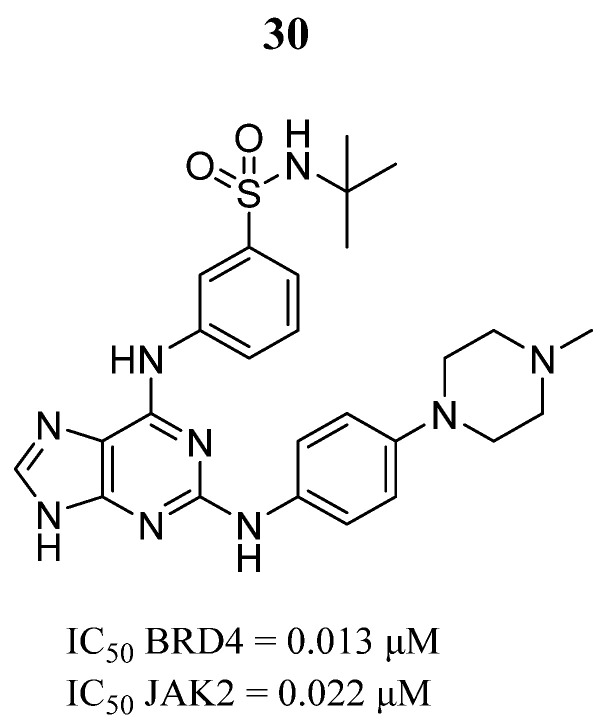
Chemical structure of the leading 9*H*-purine-2,6-diamine derivative obtained by Guo et al. [80]. In the figure, the number in bold refers to the compound described in the text, indicating its inhibitory values in BRD4 and JAK2 proteins. The figure was drawn and labeled with ChemDraw v. 19.0.

On the path of a dual compound design, Guo et al. [80] designed, synthesized, and evaluated a series of 9*H*-purine-2,6-diamine derivatives with the ability to inhibit JAK2 and bromodomain-containing protein 4 (BRD4). Compound **30** showed inhibitory activity of the bromodomain BD2 of BRD4 and JAK2 (Figure 21). The Western blot assay demonstrated that compound **30** had suppressor effects in the NF-κB signaling pathway and the phosphorylation signals of p65, IκB-α, and IKKα/β on the RAW264.7 cell line. Theoretical binding simulations showed that pyrimidine N and the ortho amino group of compound **30** formed hydrogen bonds with the Leu932 amino acid in the JAK2 hinge region. Another hydrogen bond was formed between the residue Glu930 and the purine NH group. The binding mode of compound **30** with BRD4 (BD2) showed that the partial groups—in particular, the phenyl group—formed hydrogen bonds with Pro375, Lys378, Asp381, Tyr390, and Asn433 in the cavity. Therefore, compound **30** could be a dual-target inhibitor to aid in the treatment of myeloproliferative neoplasms.

**Figure 22:**
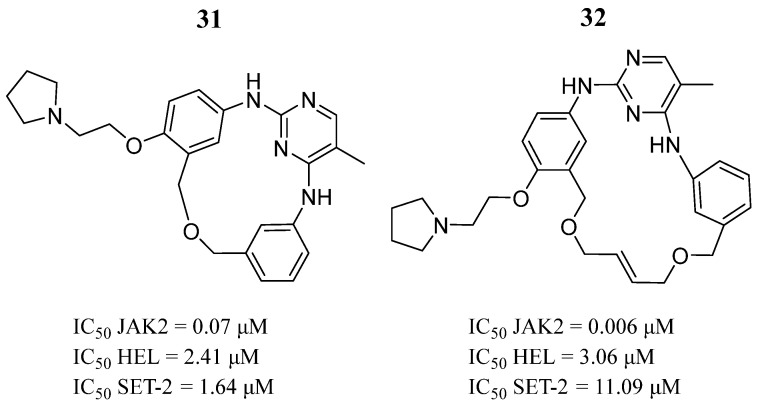
Chemical structures of the macrocycles synthesized by Diao et al. [81]. In the figure, the numbers in bold refer to the compounds described in the text, indicating their inhibitory values in JAK2 and distinct cancer cell lines. Figures were drawn and labeled with ChemDraw v. 19.0.

Diao et al. [81], using the acyclic scaffold of Fedratinib, designed new macrocyclic JAK2 inhibitors. Among 218 new macrocycles generated with different linkers, they selected three compounds for chemical synthesis. Compounds **31** and **32** demonstrated JAK2 enzymatic inhibition (Figure 22). Compounds **31** and **32** also suppressed the proliferation of the HEL and SET cell lines (Figure 22). Western blot analysis showed that compounds **31** and **32** blocked phosphorylation at the Tyr221 amino acid in HEL cells in a dose-dependent manner, which is required for the activation of JAK2. Both compounds also impaired the STAT3 and STAT5 molecules.

**Figure 23:**
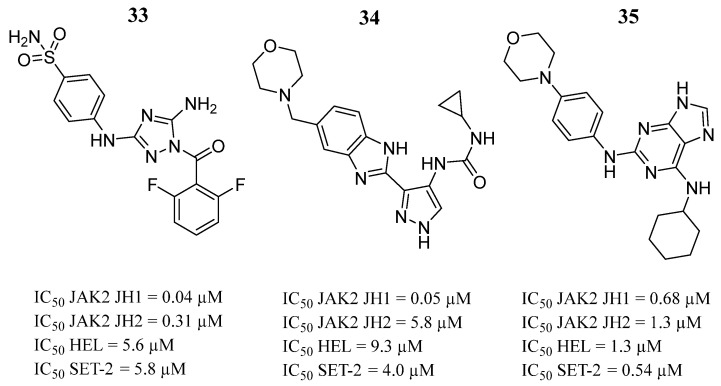
Compounds **33**, **34**, and **35** evaluated by Virtanen et al. [28]. In the figure, the numbers in bold refer to the compounds described in the text, indicating their inhibitory values in JAK2 and distinct cancer cell lines. Figures were drawn and labeled with ChemDraw v. 19.0.

Virtanen et al. [28] searched for inhibitors targeting the ATP-binding site in JH2 of V617F JAK2. Based on the binding strength to JAK2, JH2 and/or the selectivity of JH2 over JH1, they selected and evaluated eight compounds. Compounds **33**, **34**, and **35** showed the best binding values, and compounds **33** and **34** presented a highly selective binding for JH1 over JH2 in JAK2 (Figure 23), while the selectivity for JH1 of compound **35** was lower (IC_50_ = 1.3 µM over JH2) (Figure 23). Compounds **33**, **34**, and **35** also inhibited the viability of the HEL and SET-2 cell lines (Figure 23). The structural analysis showed that the compounds were anchored by hydrogen bonds in the hinge region, interacting with residues Gln626, Val629, and Glu627. Except for compound **33**, in the V617F mutant structure, it also formed a hydrogen bond with the Lys640 side chain of the αD helix. This interaction was not present in the WT structure.

**Figure 24:**
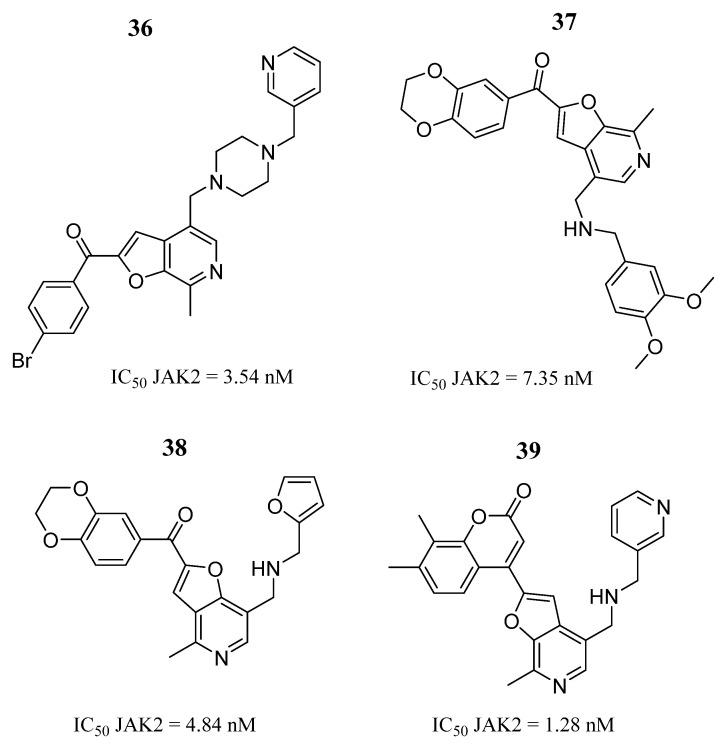
Chemical structures of the selected compounds based on furopyridine. In the figure, the numbers in bold refer to the compounds described in the text, indicating their inhibitory values in JAK2. Figures were drawn and labeled with ChemDraw v. 19.0.

Recently, Suriya et al. [82] performed a virtual screening based on the binding of 63 internal furopyridine-based compounds to the ATP-binding site of JAK2. They selected a group of six compounds for biological evaluations. The results showed that only compounds **36**, **37**, **38**, and **39** showed inhibitory activity against JAK2 in the nanomolar scale range (Figure 24), such as the drug tofacitinib (IC_50_ = 17.66 nM). In addition to enzymatic assays, the compounds were also evaluated by immunoblotting. Compound **38** showed cytotoxic effects with IC_50_ values of 57.27 and 27.28 μM in human erythroblast cell lines, respectively. Compound **37** also exhibited a cytotoxic effect on TF-1 with an IC_50_ value of 83.47 μM by suppressing JAK2/STAT5 autophosphorylation. To investigate the atomistic binding mechanisms, they carried out molecular dynamics simulations, revealing that compounds **37** and **38** bind stably to JAK2. The compounds interacted with crucial amino acids (Phe860, Gly861, Ala880, Lys882, Lys883, Leu884, Phe895, Val911, and Leu927), including glycine (Met929 and Gly935), catalytic (Gly980), and activation (Gly996 and Pro1013), as revealed by the MM/GBSA method.

**Figure 25:**
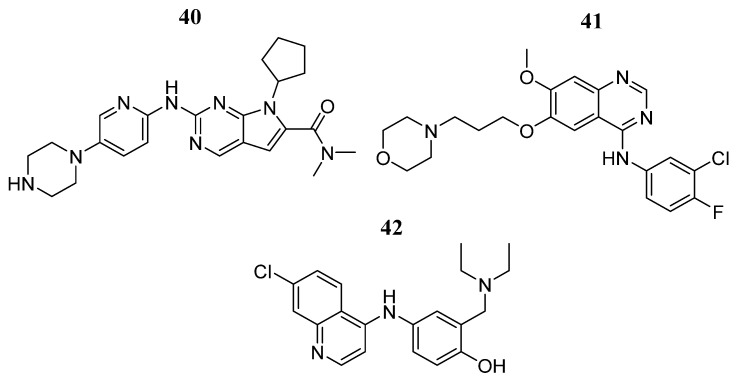
Chemical structures selected as potential JAK2 inhibitors through drug repositioning by Yasir et al. [83]. In the figure, the numbers in bold refer to the compounds described in the text. Figures were drawn and labeled with ChemDraw v. 19.0.

Yasir et al. [83] employed a graph neural network algorithm to screen a FDA-approved drug library to identify potential JAK2 inhibitors. From the virtual screening, compounds **40, 41**, and **42** (−58.0, −50.6, and −44.4 Kcal/mol) (Figure 25) were ranked among the top 10 docked compounds, exhibiting a higher negative CDocker interaction energy than the reference compound tofacitinib (−40.0 Kcal/mol). The formation of hydrogen bonds with key amino acids at the JAK2 active site dominates the binding patterns of the three compounds. In vitro analysis showed a significant JAK2 inhibition at 25 nM (between 42 and 80%) for the three selected compounds, suggesting new promising JAK2 inhibitors.

## 5. Natural Derived JAK2 Inhibitors

**Figure 26:**
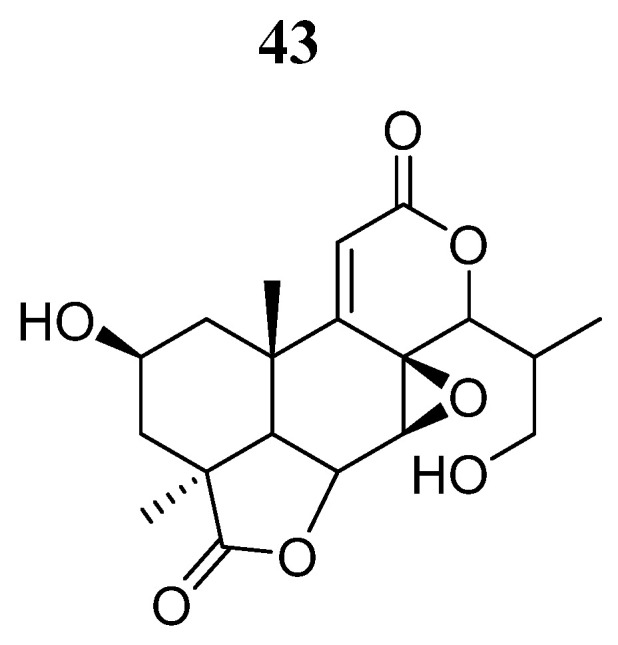
Chemical structure of 3-deoxy-2β, 16-dihydroxynagilactone E (**43**). In the figure, the number in bold refers to the compound described in the text. The figure was drawn and labeled with ChemDraw v. 19.0.

Studies have also been carried out with natural compounds such as 3-deoxy-2β,16-dihydroxynagilactone E (**43**) (Figure 26) [84]. Shan et al. [85] examined the effects of the nagilactone compound **43** on JAK family proteins. Compound **43** was demonstrated to be an allosterically inhibitor that interacts with the FERM-SH2 domain of JAK2, which also leads to the inhibition of STAT3 phosphorylation. In MDA-MB-231 and MDA-MB-468 breast cancer cells, compound **43** inhibited growth and induced apoptosis through impairment of STAT3 signaling.

**Figure 27:**
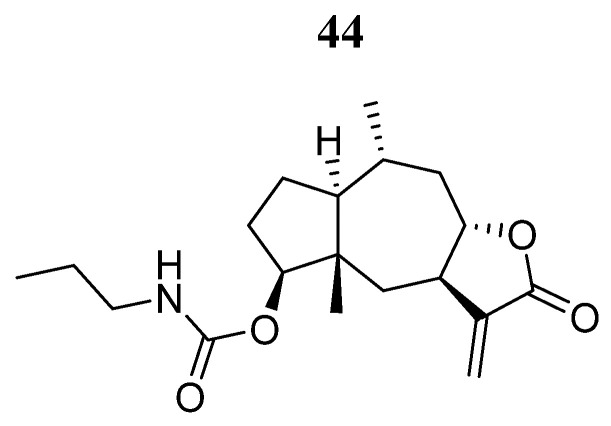
Chemical structure of 2-deoxy-4β-propylcarbamate-pulchelin (**44**). In the figure, the number in bold refers to the compound described in the text. The figure was drawn and labeled with ChemDraw v. 19.0.

Another natural compound studied is 2-deoxy-4β-propylcarbamate-pulchelin (**44**) (Figure 27), a sesquiterpene lactone derived from 2-deoxy-4-epi-pulchelin from the medicinal herb *Carpesium abrotanoides* L. Huang et al. [86] showed that compound **44** inhibits the growth of a panel of human cancer cell lines (A375, A2058, DU145, H4, BEL7404, LM3, MDA-MB-453, MDA-MB-231, HT29, HCT116, HT1650, and H460) with IC_50_ values ranging from 3.0 to 21.0 μM and also inhibited JAK2 kinase activity. However, the inhibitory effects of compound **44** can be blocked by the reducing agents dithiothreitol or glutathione, indicating the importance of the thiol-reactive α-β unsaturated carbonyl group. Mass spectrography and molecular docking analysis revealed that compound **44** bound covalently to the Cys452 amino acid in the SH2 domain. Compound **44** could also form noncovalent interactions with His451, Phe439, Lys464, Lys454, Lys453, and Phe436 amino acids.

**Figure 28:**
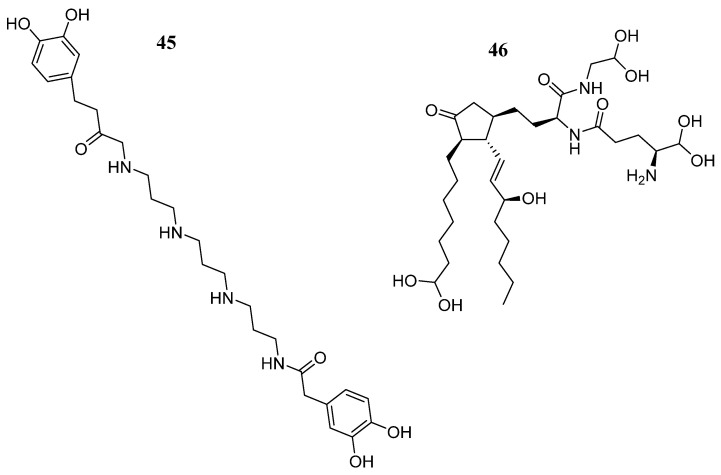
Chemical structure of compounds **45** and **46** selected from a virtual screening by Li W et al. [75]. In the figure, the numbers in bold refer to the compounds described in the text. Figures were drawn and labeled with ChemDraw v. 19.0.

Li et al. [71] downloaded 17,799 natural product compounds from the ZINC15 database. After virtual screening, they found that 667 compounds had better binding libdock scores than the reference ligand Fedratinib (libdock score: 129.056) on JAK2. Compounds **45** and **46** (Figure 28) showed the highest binding affinities (−62.46 and −56.61 Kcal/mol, respectively). Compound **45** formed hydrogen bonds with Lys943, Leu855, and Leu932; carbon hydrogen bonds with Gly856, Asp939, and Lys857; one pi-pi interaction (Tyr931); and four Pi-alkyl bond interactions (Leu983, Val863, Ala880, and Leu855) with JAK2. Compound **46** formed hydrogen bonding interactions with Lys882, Arg980, Ser936, Asp939, and Lys943; carbon hydrogen bonding interactions with Leu932 and Leu855; and a sulfur bonding interaction with Lue855 on JAK2. Further predictions showed that these compounds had non-Ames mutagenicity, low carcinogenicity in rodents, lower potential for developmental toxicity, and no liver toxicity. Molecular dynamics simulation demonstrated that these two complexes could stably exist under natural circumstances, making them promising drugs in the treatment of patients with primary myelofibrosis.

**Figure 29:**
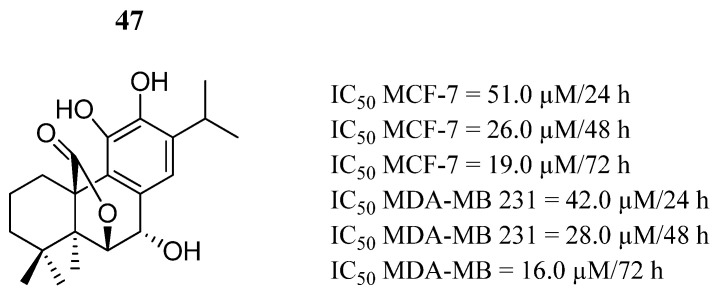
Chemical structure of Rosmanol (**47**). In the figure, the number in bold refers to the compound described in the text, indicating its inhibitory values in distinct cancer cell lines. The figure was drawn and labeled with ChemDraw v. 19.0.

Jiang et al. [87] investigated the effects of the phenolic diterpene compound **47**, known as Rosmanol (Figure 29). They showed that compound **47** had anti-proliferation effects on MCF-7 and MDA-MB 231 breast cancer cells (Figure 29) but did not have a significant effect on the normal MCF-10A breast cell line. Compound **47** also promoted an apoptotic process associated with alterations in mitochondrial pathways, the production of reactive oxygen species (ROS), and cell cycle arrest in phase S. In MCF-7 and MDA-MB 231 cells, compound **47** induced increased expression of PIAS3 in a dose-dependent manner. Compound **47** also decreased the level of phosphorylated STAT3; therefore, they demonstrated that compound **47** inhibited STAT3 phosphorylation and transcription activity by increasing PIAS3 or inhibiting JAK2 phosphorylation.

**Figure 30:**
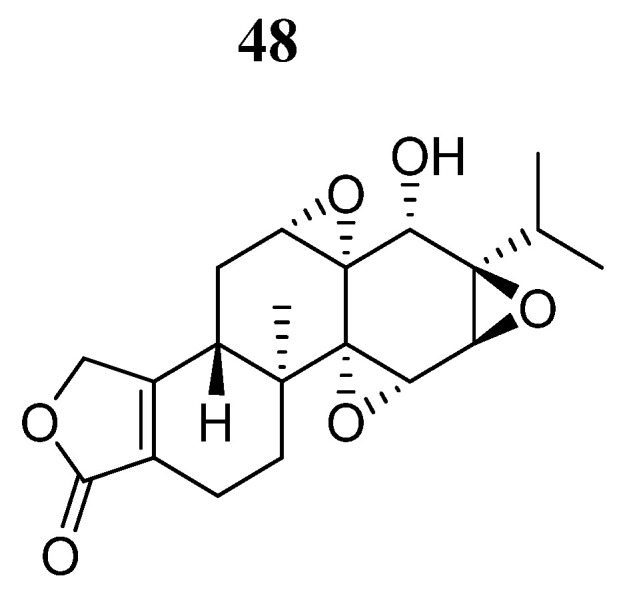
Chemical structure of triptolide (**48**). In the figure, the number in bold refers to the compound described in the text. The figure was drawn and labeled with ChemDraw v. 19.0.

Another natural compound (**48**), the triptolide TLP (Figure 30) with known anticancer and inflammatory effects on cisplatin (DDP)-resistant ovarian cancer [88,89], was evaluated by Zhong et al. [90] in the context of the autophagic role of SKOV3/DDP cells derived from DDP-resistant ovarian cancer. It was shown that TPL induced autophagy, facilitating the death of SKOV3/DDP ovarian cancer cells, and was associated with ROS generation and inhibition of the JAK2/STAT3 pathway. However, the inhibitory effect of compound **48** on the JAK2/STAT3 pathway could be restored in the presence of the antioxidant N-acetyl-L-cysteine. Furthermore, compound **48** changes the interaction between Mcl-1 and Beclin1 but can be avoided by IL-6 stimulation.

**Figure 31:**
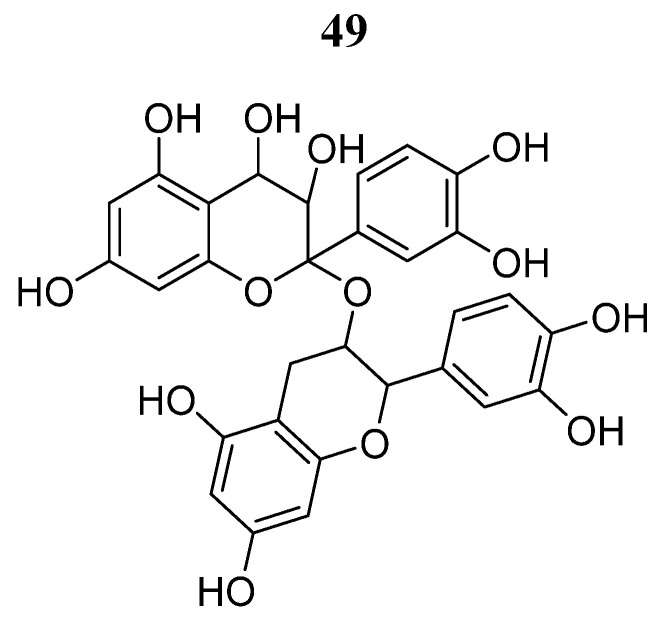
Chemical structure of proanthocyanidins (**49**). In the figure, the number in bold refers to the compound described in the text. The figure was drawn and labeled with ChemDraw v. 19.0.

Other polyphenolic compounds found in plants are proanthocyanidins (**49**) (Figure 31). Wu et al. [91] studied the effects of this natural product on A549 lung cancer cells. Compounds **49** showed cell antiproliferative effects and changed the cloning ability of A549 cells (IC_50_ of 150.7 and 78.41 µM, respectively). Furthermore, it inhibited migration and invasion in a dose-dependent manner in the Transwell chamber assay. At the same time, it induced apoptosis and cell cycle arrest in the G2/M phase. Furthermore, this compound acts synergistically with the AG490 JAK2/STAT3 inhibitor to stop A549 cell invasion and migration. These results demonstrated that **49** could mediate the antitumor effect of non-small cell lung cancer through the JAK2/STAT3 pathway.

**Figure 32:**
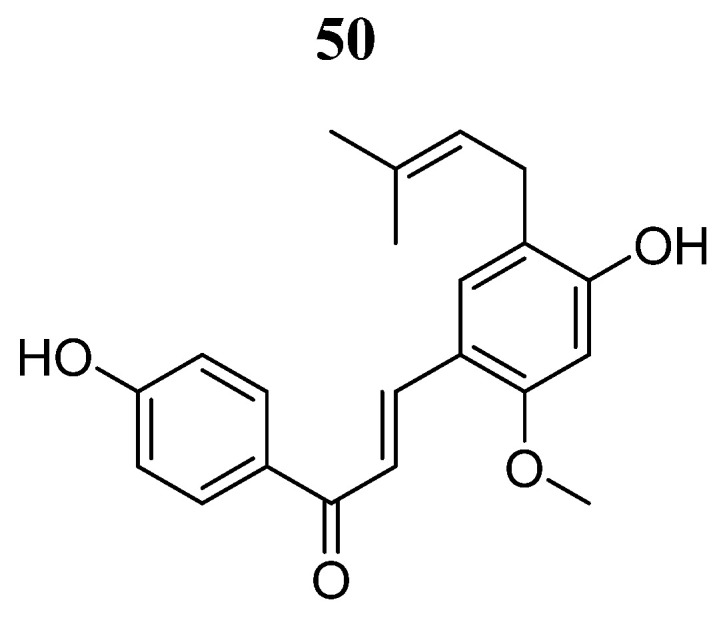
Chemical structure of licochalcone H (**50**). In the figure, the number in bold refers to the compound described in the text. The figure was drawn and labeled with ChemDraw v. 19.0.

Licochalcone H (**50**) (Figure 32) derived from licochalcone C was evaluated by Park et al. [92] on A375 and A431 human skin cancer cells. Phenolic compound **50** inhibited cell growth in a dose- and time-dependent manner, induced apoptosis, and led to cell cycle arrest in phase G1. Furthermore, the phosphorylation of JAK2 and STAT3 phosphorylation was decreased. The inhibitory effects of this compound were similar to those produced by cryptotanshinone and S3I-201 JAK2/STAT3 signaling inhibitors.

**Figure 33:**
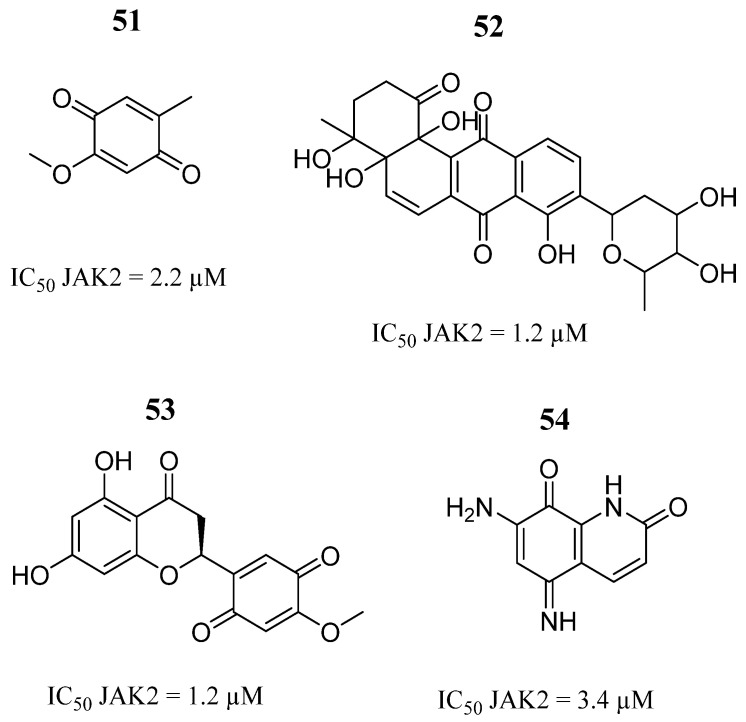
Chemical structure of the natural products evaluated by Pölläniemi et al. [7]. In the figure, the numbers in bold refer to the compounds described in the text, indicating their inhibitory values in JAK2. Figures were drawn and labeled with ChemDraw v. 19.0.

Pölläniemi et al. [7] performed an optimized assay to select a library of natural products against the JAK2 kinase domain and compared the performance of the assay with differential scanning fluorimetry. Using the fluorescence polarization (FP) tracer shift assay, coprinin (**51**), acuaimicin (**52**), NSC169517 (**53**), and NSC111041 (**54**) (Figure 33) were selected for dose-response measurements. The four compounds showed micromolar inhibitory effects against the JAK2 enzyme (Figure 33). Among them, compounds **51** and **52** inhibited the kinase activity at a concentration of 10 μM (43 and 55%). On the other hand, compound **54** was identified as a competitive inhibitor with IC_50_ = 21 µM in the FP assay.

**Figure 34:**
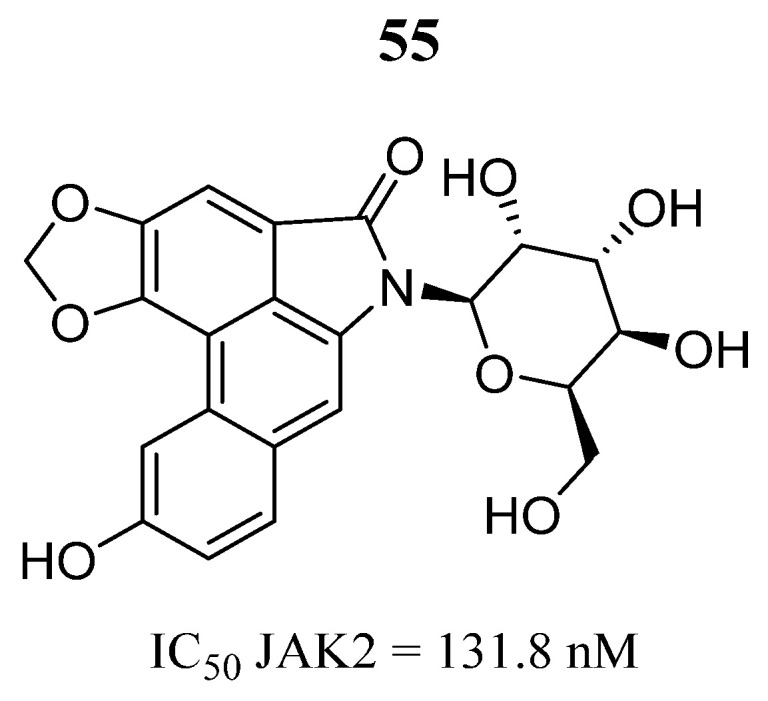
Chemical structure of compound **55** identified through high-throughput virtual screening as a potential JAK2 inhibitor by Shaikh et al. [93]. In the figure, the number in bold refers to the compound described in the text, indicating its inhibitory value in JAK2. The figure was drawn and labeled with ChemDraw v. 19.0.

Shaikh et al. [93] identified a natural product (**55**) (Figure 34) from the ZINC database using simulations based on the structure of JAK2. This compound interacts with the amino acid residues Ala880, Val863, Tyr931, Leu855 Asp994, Leu983, Asn981, and Arg980, which were implicated with the predicted binding energy of −175.43 Kcal/mol. Kinase inhibition assays show that compound **55** inhibits JAK2 kinase in the nanomolar range (Figure 34). The results suggest that compound **55** could be a suitable natural product that targets JAK2.

**Figure 35:**
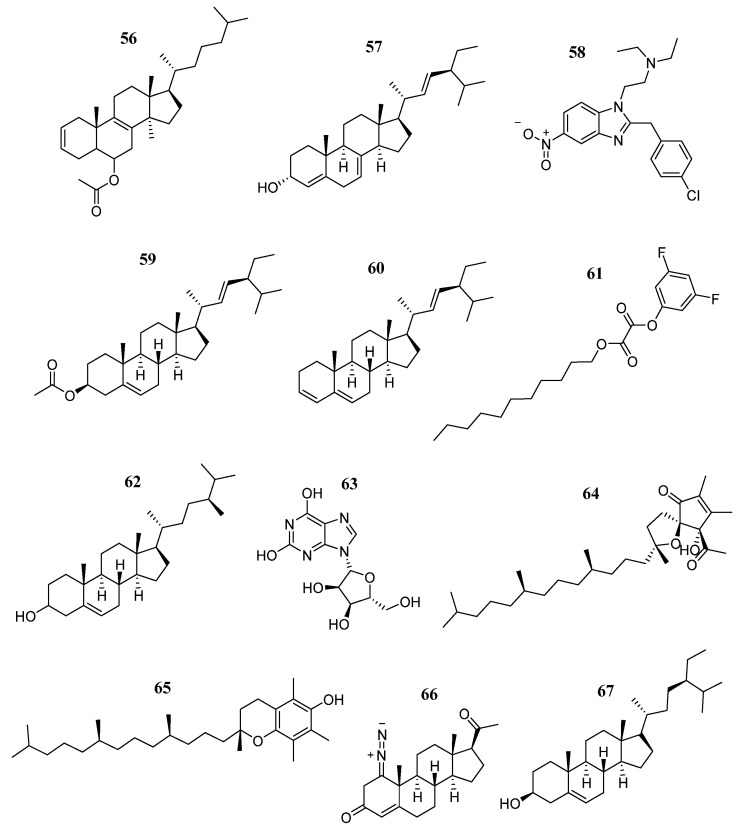
Chemical structures of compounds obtained from natural products (Upreti et al.) [94]. In the figure, the numbers in bold refer to the compounds described in the text. Figures were drawn and labeled with ChemDraw v. 19.0.

Upreti et al. [94] collected leaf and stem samples from *Urtica dioica* and *U. parviflora* plants to extract the most abundant metabolites using different solvents (hexane, chloroform, and ethanol). GC-MS extract analysis revealed that a total of 175 metabolites are the most abundant. Through a virtual screening, they identified 12 main compounds (**56–67**) (Figure 35) with a binding energy of −8.6 to −7.1 Kcal/mol that was equal to or better than Paclitaxel control (−7.1 Kcal/mol). The interaction analysis showed that all 12 compounds showed interaction patterns with the Leu983, Leu855, Val863, Arg980, Val863, Ala880, Leu855, and Leu932 amino acids of the binding pocket of JAK2. Furthermore, the whole extracts obtained from *Urtica* spp. showed anticancer activity against MDA-MB-231 (TNBC cell line) (IC_50_ 90.09 to 172.16 μg/mL) and lower cytotoxicity in healthy cell lines (HEK293T) (IC_50_ of 732.52 to 1367.25 μg/mL).

**Figure 36:**
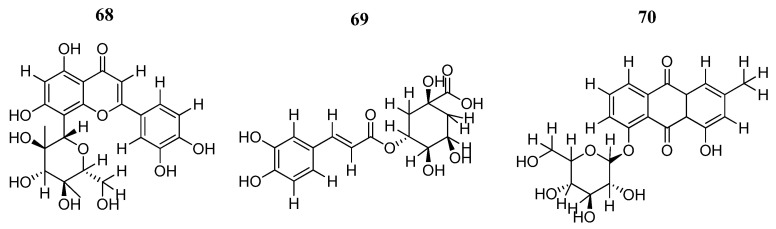
Chemical structures of the natural compounds analyzed by Vaziri-Amjad et al. [95]. In the figure, the numbers in bold refer to the compounds described in the text. Figures were drawn and labeled with ChemDraw v. 19.0.

Vaziri-Amjad et al. [95] also worked with natural compounds using computational techniques for drug discovery. Of 79 plant-derived metabolites, twelve flavonoids, two anthraquinones, and three cinnamic acid derivatives had the best predicted binding affinities (lower than −10.0 Kcal/mol) at the JAK2 ATP-binding site. Flavonoid orientin (**68**), chlorogenic acid derived from cinnamic acid (**69**), and the anthraquinone pulmatin (**70**) (Figure 36) showed binding affinities of −14.49, −11.87, and − 10.76 Kcal/mol, respectively. Molecular dynamics simulations showed stability for the three compounds, with average RMSD values of 2.04 (**68**), 2.06 Å (**69**), and 1.95 Å (**70**), respectively. MM-GBSA calculations provided binding energy estimates, ranking compound **69** as the strongest binder (−24.13 Kcal/mol), followed by compound **68** (−21.28 Kcal/mol) and then compound **70** (−14.76 Kcal/mol). Compound **70** formed the largest number of hydrogen interactions with JAK2 with seven hydrogen bonds, compound **69** with four hydrogen bonds, and compound **68** with one hydrogen bond.

## 6. JAK3 Inhibitors

**Figure 37:**
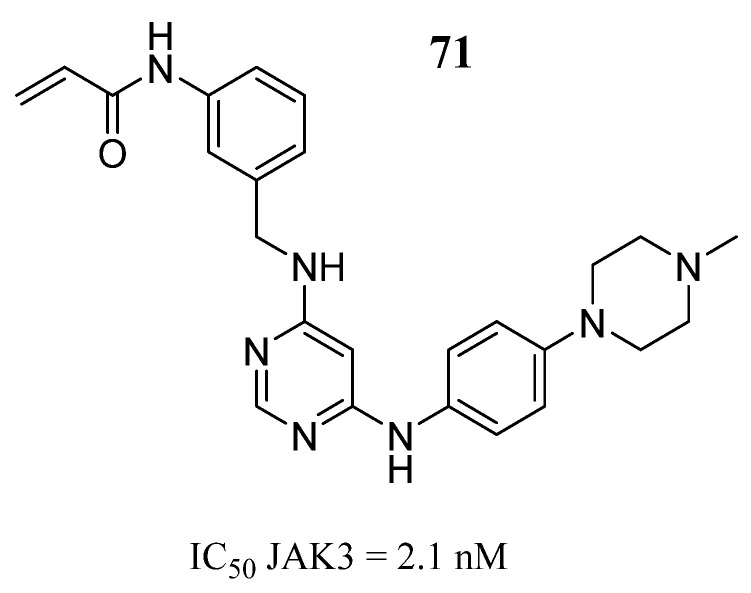
Chemical structure of the pyrimidine-4,6-diamine derivative as a selective JAK3 inhibitor described by Yu et al. [96]. In the figure, the number in bold refers to the compound described in the text, indicating its inhibitory value in JAK3. The figures was drawn and labeled with ChemDraw v. 19.0.

Yu et al. [96] designed pyrimidine-4,6-diamine derivatives as selective JAK3 inhibitors. Compound **71** was found to exhibit JAK3 inhibitory activity (Figure 37) and JAK kinase selectivity over JAK1/2 and TYK2 (IC_50_ of 945 to >3000 nM). Molecular docking study predicted that the pyrylamine group forms bidentate hydrogen bonds with Leu905, and the Cys909 in JAK3 has been attacked by the acrylamide group, since the distance between the sulfur atom of Cys909 and the acrylamide warhead in compound **71** was 1.94 Å. The side chain of compound **71** extends into the hydrophobic region. Furthermore, the phenyl group of compound **71** had van der Waals contact with Leu956 and Leu828 in the ATP-binding pocket. Additionally, acrylamide forms a hydrogen bond with the carbonyl of Arg953. In the cellular assay, **71** showed moderate potency in inhibiting the proliferation of IL-2 stimulated T cells (IC_50_ = 12 nM). The authors observe that the introduction of a piperazine ring and an acrylamide group improves the affinity for JAK3.

**Figure 38:**
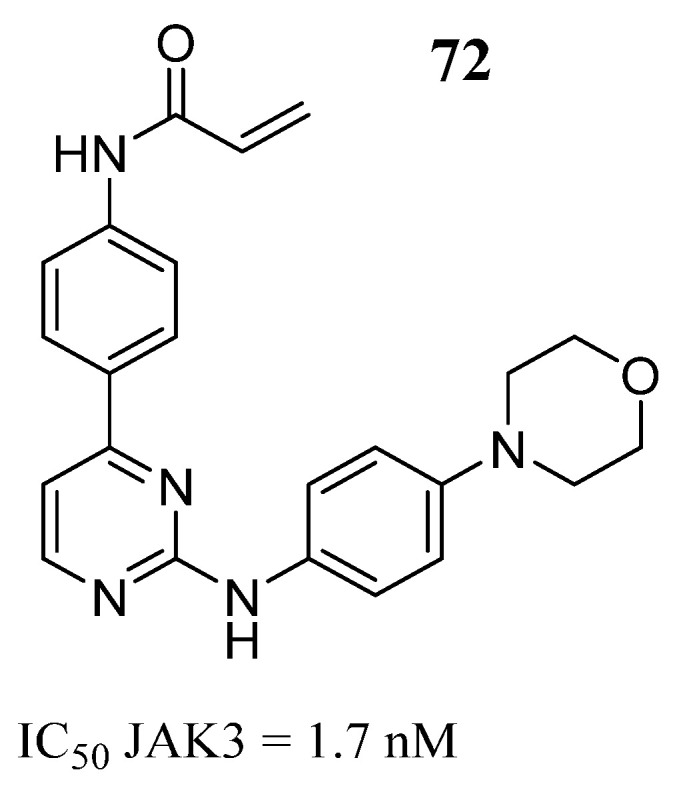
Chemical structure of the phenylpyrimidine derivative analyzed by Shu et al. [20] as a potential JAK3 inhibitor. In the figure, the number in bold refers to the compound described in the text, indicating its inhibitory value in JAK3. The figures was drawn and labeled with ChemDraw v. 19.0.

Shu et al. [20] selected compounds that had little selectivity based on 4- or 6-phenylpyrimidine derivatives to convert them into more active and selective compounds by designing a covalent bond with the Cys909 residue in JAK3. Compound **72** exhibited high inhibitory activity (Figure 38) with a very good selectivity profile compared to the other JAK isoforms 588 times higher. In a cellular assay, compound **72** strongly inhibited JAK3-dependent signaling and T-cell proliferation (IC_50_ of 0.83 µM anti-CD3/CD28 stimulation; IC_50_ of 0.77 µM IL-2 stimulation). The analysis of the binding mode on JAK3 showed that the anilinopyrimidine moiety of compound **72** formed hydrogen bonds with Leu905 and a continuous electron density between the acrylamide and Cys909. The main difference in the 72 binding patterns to JAK3 was the morpholinoaniline group, which was associated with the Leu828 and Gly908 amino acids through two σ-π predicted interactions and the σ-π interaction between the pyrimidine group and the Leu828 amino acid. On the other hand, the thiol group and Asp912 formed a hydrogen bond after the acryl group and the thiol group of the Cys909 residue that formed the covalent bond, indicating that compound **72** had close contact with the ATP-binding pocket in JAK3. Thus, the molecular docking analysis helped the authors to explain the in vitro results.

**Figure 39:**
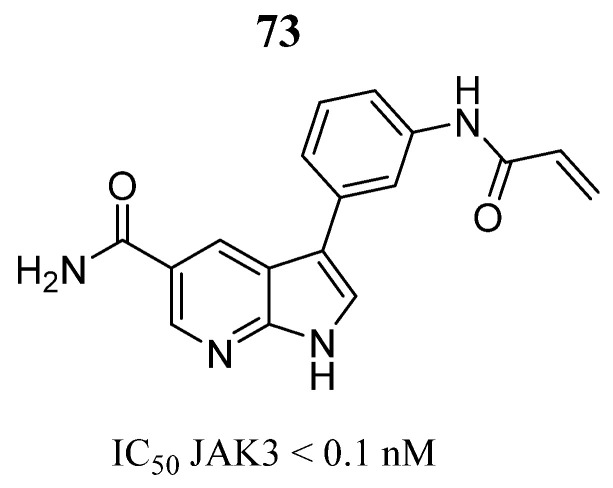
Chemical structure of the 1*H*-pyrrolo [2,3-*b*]pyridine derivative evaluated on JAK3 by Forster et al. [17]. In the figure, the number in bold refers to the compound described in the text, indicating its inhibitory value in JAK3. Figures were drawn and labeled with ChemDraw v. 19.0.

Forster et al. [17] synthesized 1*H*-pyrrolo [2,3-*b*]pyridine derivatives and evaluated them enzymatically; compound **73** had the best exhibiting activity in JAK3 with IC_50_ values in the nanomolar range (Figure 39) with selectivity values of IC_50_ < 460 nM against other JAKs. While molecular docking analysis showed that the 7-azaindole group was attached by two hydrogen bonds by the Lys905 located in the hinge region and also had a hydrogen bond between a NH carboxamide and the carbonyl group of the Glu903 residue. Furthermore, the amide group was involved in the water-mediated hydrogen bonding with the Cys909 residue, which was suggested to help the orientation of the Michael acceptor to facilitate the addition of cysteine in the covalent bonding.

**Figure 40:**
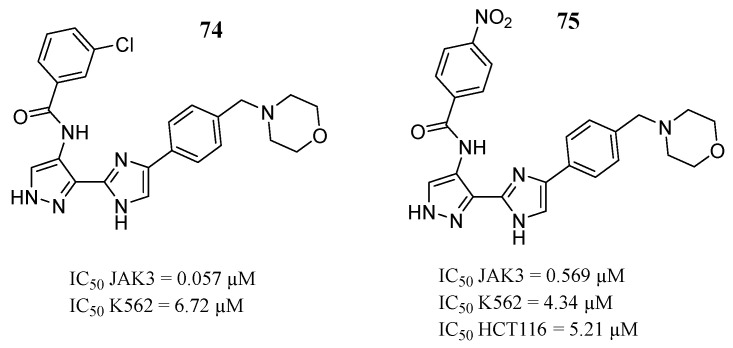
Chemical structure of 3-(4-phenyl-1*H*-imidazol-2-yl)-1H-pyrazole derivatives synthesized and evaluated by Zheng et al. [97]. In the figure, the numbers in bold refer to the compounds described in the text, indicating their inhibitory values in JAK3 and distinct cancer cell lines. Figures were drawn and labeled with ChemDraw v. 19.0.

Zheng et al. [97] synthesized and evaluated a series of 3-(4-phenyl-1*H*-imidazole-2-yl)-1*H*-pyrazole derivatives to determine their in vitro activities. In enzymatic assays, compounds **74** and **75** were more selective against JAK3 (Figure 40) than against JAK2 (IC_50_ = 0.166 and 1.178 µM, respectively). Furthermore, compound **75** showed cytotoxicity against K562 and HCT116 cells (Figure 40). While compounds **74** expressed antiproliferative activities against K562 (Figure 40). They selected compound **74** for Western blot analysis, where compound **74** negatively regulated STAT3 and STAT5 phosphorylation in a dose-dependent manner in K562 and HCT116 cells. Furthermore, compound **74** inhibited cell proliferation by inducing arrest of the G2 phase cell cycle. Molecular modeling predicts that compound **74** might interact with JAK2 through five amino acid residues at the binding site (Glu930, Tyr931, Leu932, Ser936, and Gly993). While compound **75** in JAK3 presented three interactions with amino acid residues Glu903, Tyr904, and Leu905.

**Figure 41:**
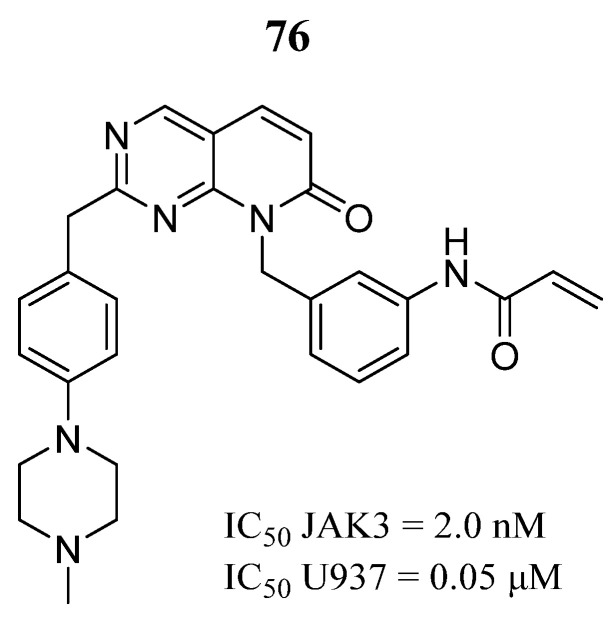
Chemical structure of the pyrido [2,3-*d*]pyrimidin-7-one derivative synthesized as a potential JAK3 inhibitor by Su et al. [21]. In the figure, the number in bold refers to the compound described in the text, indicating its inhibitory value in JAK3 and a cancer cell line. The figure was drawn and labeled with ChemDraw v. 19.0.

Su et al. [21] reported the synthesis of a series of covalent JAK3 inhibitors with a scaffold of pyrido [2,3-d] pyrimidin-7-one. Compound **76** was found to be the most potent inhibitor of JAK3 (Figure 41) and selectivity over JAK1/2 and TYK2 (IC_50_ = 2434, 1131, and 8877 nM, respectively). Furthermore, it showed inhibitory activity against U937 cells derived from diffuse histiocytic lymphoma that express the JAK3 M511I mutation. Compound **76** also inhibited the phosphorylation of JAK3 and STAT5 signaling in U937 cells.

**Figure 42:**
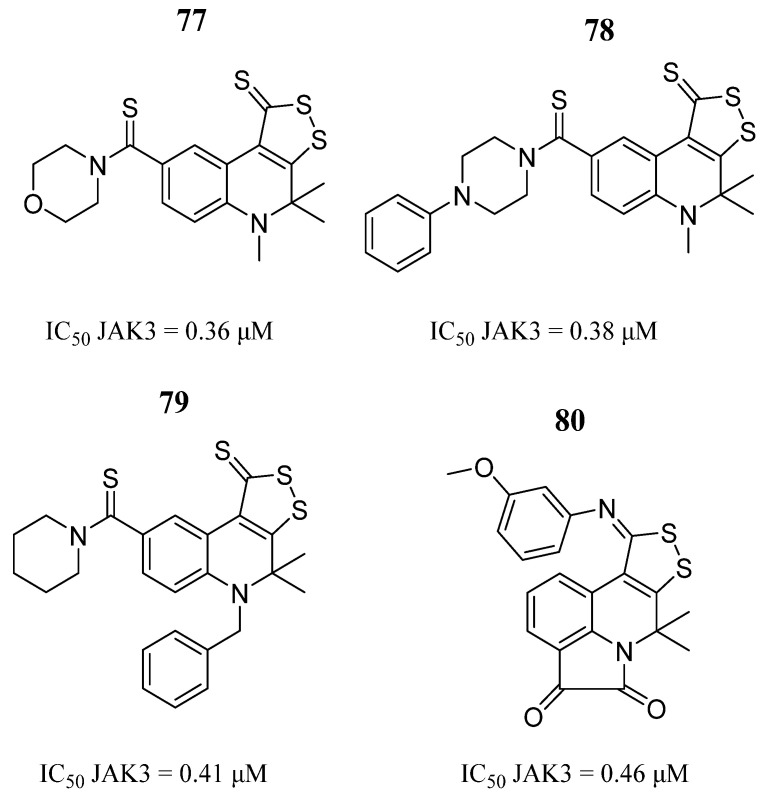
Chemical structure of hybrid and chimeric derivatives of 4,5-dihydro-4,4-dimethyl-1*H*-[1,2]dithiolo [3,4-*c*]quinoline-1-thiones obtained as potential JAK3 inhibitors by Medvedeva et al. [98]. In the figure, the numbers in bold refer to the compounds described in the text, indicating their inhibitory values in JAK3 Figures were drawn and labeled with ChemDraw v. 19.0.

Medvedeva et al. [98] synthesized a set of 4,5-dihydro-4,4-dimethyl-1*H*-[1,2]dithiolo[3,4-*c*]quinolin-1-thiones hybrid 4,5-dihydro-4,4-dimethyl-1*H*-[1,2]dithiolo[3,4-c] quinolin-1-thiones containing the tricyclic fragment joined with other heterocyclic fragments. PASS online-based predictions for 1,2-dithiolo [3,4-*c*]quinoline-1-thione derivatives identified twelve compounds with chemoprotective and antitumor activity. Compounds **77**, **78**, **79**, and **80** showed a significant inhibition of JAK3 in in vitro studies (Figure 42).

**Figure 43:**
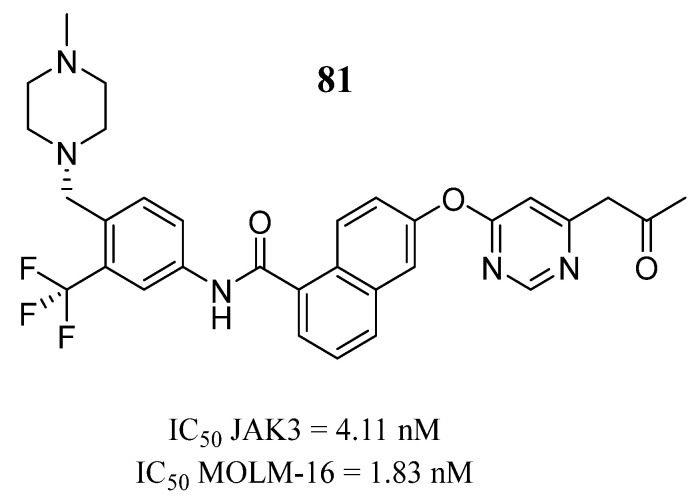
Chemical structure of the potential JAK3 inhibitor identified by structure-based high-throughput virtual screening by Wei et al. [99]. In the figure, the number in bold refers to the compound described in the text, indicating its inhibitory values in JAK3 and cancer cell lines. The figures was drawn and labeled with ChemDraw v. 19.0.

Wei et al. [99] carried out a high-performance hybrid structure-based virtual sensing protocol using deep learning algorithms, molecular docking, and molecular dynamics simulations to guide the selection of potential JAK3 for further in vitro evaluation. The results showed that compound **81** presented inhibitory potency against JAK3 and in the MOLM-16 cell line (Figure 43). Compound **81** could have hydrogen bond interactions with Lys855 and Arg953 after induced-fit docking. In the molecular dynamics study, they observed that the nitrogen atom of the pyrimidine residue could generate a strong hydrogen bond interaction with Lys855. Furthermore, the oxygen atom of the amide formed an additional hydrogen bond with Leu905. Meanwhile, the –CF3 group had hydrophobic interactions with the amino acids Leu905, Gly906, and Arg953 that could be related to the inhibitory activity against JAK3.

**Figure 44:**
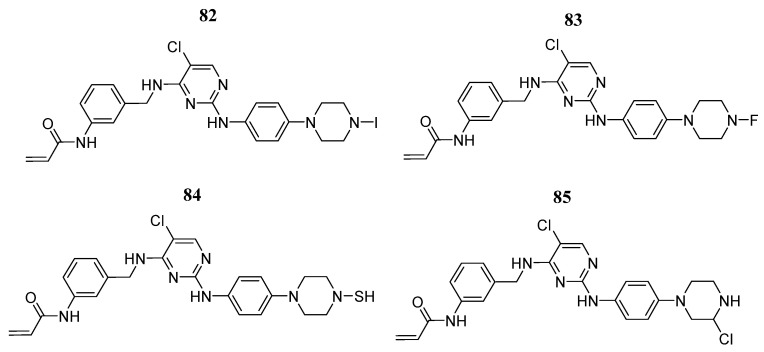
Chemical structures of compounds **82–85** identified as potential JAK3 inhibitors using predictive techniques by Faris et al. [100]. In the figure, the numbers in bold refer to the compounds described in the text. Figures were drawn and labeled with ChemDraw v. 19.0.

Faris et al. [100] built and validated a prediction model using Monte Carlo methods to identify four compounds (**82**, **83**, **84**, and **85**) (Figure 44) that exhibited strong pIC_50_ values (9.90 to 9.59) in the enzymatic studies. The molecular docking simulations showed that compounds **82**, **83**, **84**, and **85** have the best affinity scores (from −10.06 to −8.9 Kcal/mol). The binding mode analysis showed hydrogen interactions with Gln827, Leu905, and Arg953 amino acids. In addition, there were some carbon–hydrogen bonds and pi-sigma interactions. Compound **83** showed two hydrogen bonds with Leu905 and carbon–hydrogen bonds with Asp912, Leu828, and Glu903. Furthermore, it had a pi-sigma interaction with Gly908 and alkyl interactions with Ala853, Val884, Met902, Arg911, and Arg953. Similarly, it showed pi-alkyl interactions with Leu828, Ala853, Leu905, Leu956, Leu828, and Leu956. Compound **84** formed hydrogen bonds with Gln827, Leu905, and Arg953. It also formed a carbon–hydrogen bond with Leu828, Gln827, and Glu903. Furthermore, it exhibited a pi-sigma interaction with Gly908 and an alkyl interaction with Arg953. Also, it showed pi-alkyl interactions with Leu828, Ala853, Leu905, Leu956, Leu828, Leu956, and Ala966. Finally, compound **85** formed hydrogen bonds with Gln827, Leu905, and Arg953. It also formed carbon–hydrogen bonds with Gln827, Leu828, and Glu903. Furthermore, it showed a pi-sigma interaction with Gly908 and alkyl interactions with Ala853, Val884, Met902, and Arg953. Likewise, it exhibited pi-alkyl interactions with Leu828, Ala853, Leu905, Leu956, and Leu828. Molecular dynamics analysis suggested that the ligand–JAK3 complexes achieve binding stability during the simulation time.

**Figure 45:**
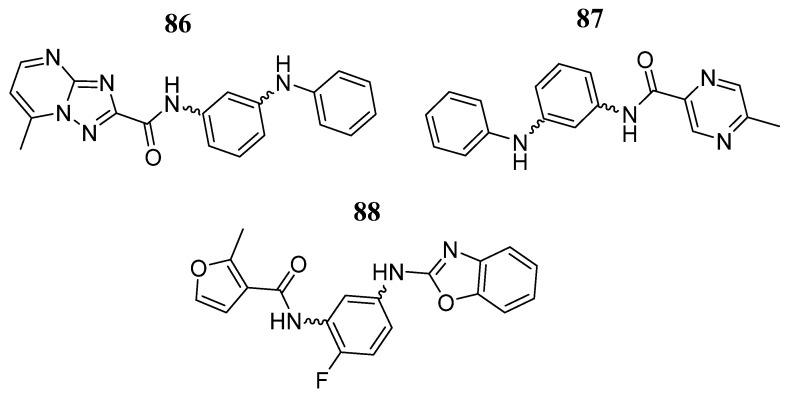
Chemical structure of the compounds selected as potential JAK3/STAT inhibitors based on the pyrimidine-4,6-diamine structure by Faris et al. [101]. In the figure, the numbers in bold refer to the compounds described in the text. Figures were drawn and labeled with ChemDraw v. 19.0.

In the same year, Faris et al. [101], based on the pyrimidine-4,6-diamine structure, employed a molecular modeling approach to design and predict new JAK3/STAT inhibitors. After generating a pharmacophore model and using the ZINCPharmer database, nine molecules were obtained as potential JAK3 inhibitors. Of these, only three molecules were selected for molecular docking analysis (**86**, **87**, and **88**) (Figure 45), with predicted plC_50_ values of 8.67, 8.57, and 8.99. In the binding mode analysis, compound **86** interacted with JAK3 through amino acid contacts with Leu905, Arg911, Arg953, Cys909, Leu828, and Ala853 and had a docking score of −6.95 Kcal/mol. The JAK3-**87** complex exhibited a docking score of −5.54 Kcal/mol and interactions with amino acids Arg953, Leu828, Cys909, Leu956, and Ala853. Compound **88** had a docking score of −9.47 Kcal/mol and had interactions with Asp912, Leu905, Cys909, Arg911, Gly908, Met902, Val836, Val884, Leu828, and Ala853. In the 500 ns molecular dynamics simulation, the three compounds showed stability with average RMSD values of 3.05, 2.18, and 2.24 Å, respectively. The maximum number of hydrogen bonds formed during the simulation was 6, 4, and 5, respectively, with a minimum of one hydrogen bond formed for all three ligands. Analysis of tofacitinib in comparison with the three compounds showed few discrepancies during 500 ns, indicating an affinity between the studied ligands and the JAK3 protein.

## 7. Natural Derived JAK3 Inhibitors

**Figure 46:**
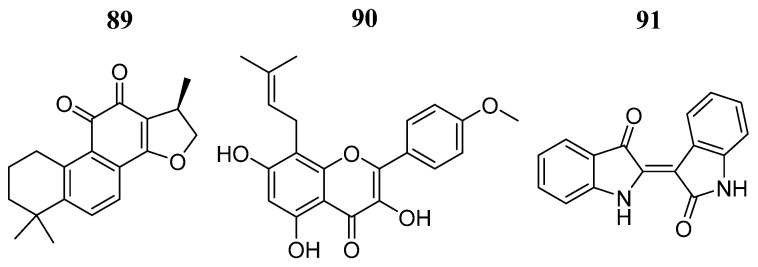
Chemical structure of Chinses herbal compounds analyzed by Su et al. [102]. In the figure, the numbers in bold refer to the compounds described in the text. Figures were drawn and labeled with ChemDraw v. 19.0.

Su et al. [102] identified three compounds, **89**, **90**, and **91** (Figure 46), of herbal origin with a demonstrated reduction in JAK3 activity (IC_50_ < 100 μM). The three compounds attenuated JAK3 phosphorylation induced by IL-2 in the HEK 293 cell line. The molecular docking analysis showed that compound **90** presented a docking score of 9.3 Kcal/mol and formed hydrogen bonds with kinase domain residues Ala966 and Asp967, including Glu903 and Leu905, while compound **91** showed a docking score of 9.1 Kcal/mol and formed hydrogen bonds with the Cys909 residue. Compound **89** had a docking score of 9.2 Kcal/mol and did not form hydrogen bonds. To investigate the stability of each complex, they studied the binding conformations of the complexes using molecular dynamics simulation tests showing a RMSD of 1.5–3 Å, demonstrating the stability of the complexes during the molecular dynamics simulations.

**Figure 47:**
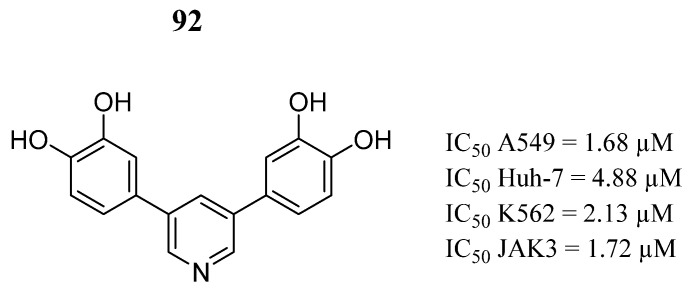
Chemical structure of natural products from *B. japanensis* analyzed by Yan et al. [103] as potential JAK3 inhibitors. In the figure, the number in bold refers to the compound described in the text, indicating its inhibitory values in JAK3 and distinct cancer cell lines. The figures was drawn and labeled with ChemDraw v. 19.0.

In the search for natural products from insects, *Blaps japanensis* attracted the attention of Yan et al. [103], where they isolated and structurally identified compounds with potential inhibitory activity against human cancer cells (A549, Huh-7, and K562) and inhibitory enzymatic activity in JAK3. They found that compound **92** was the most active against cancer cells (Figure 47). Furthermore, compound **92** also showed inhibitory activity against JAK3 (Figure 47). These findings reveal the structural diversity of the medicinal products derived from insects.

**Figure 48:**
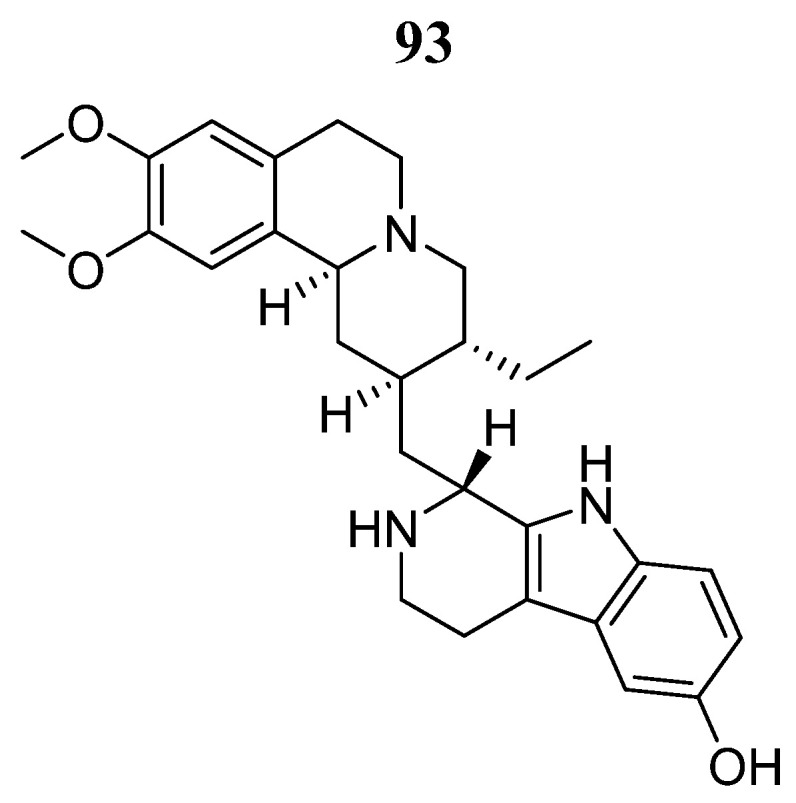
Chemical structure of tubulosin (**93**). In the figure, the number in bold refers to the compound described in the text. Figures were drawn and labeled with ChemDraw v. 19.0.

Kim et al. [104], through a JAK3 structure-based computational, identified compound **93** (Figure 48) as a potential inhibitor. Molecular docking predicted a binding score of −11.79 Kcal/mol and interactions with the amino acids Val812, Ala829, Glu847, Met878, Leu881, Leu932, and Asp943. Compound **93** decreased JAK3 activity in the presence of ATP in a concentration-dependent manner with an IC_50_ value of 9.9 nmol/L. Furthermore, compound **93** inhibited the other members of the JAK family but with a lower degree of inhibition than for JAK3, with IC_50_ values of 69.5, 84.9, and 76.3 nmol/L for JAK1, JAK2, and TYK2, respectively. Furthermore, compound **93** without modifying other oncogenic signals affected the survival and proliferation of several cancer cells (L540, BKO-84, and BaF3/JAK3V674A), inducing apoptotic and necrotic/autophagic cell death.

## 8. Dual JAK2/3 Inhibitors

**Figure 49:**
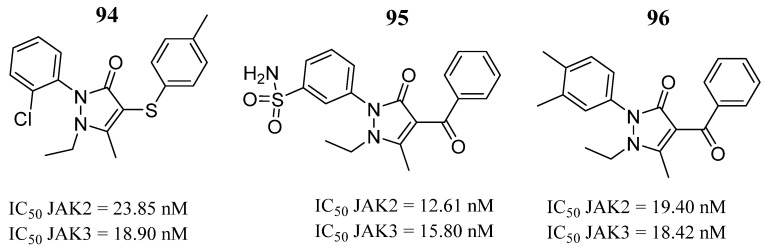
Chemical structure of the compounds selected by Sanachai et al. [49] from pharmacophore models built from the commercial drug tofacitinib. In the figure, the numbers in bold refer to the compounds described in the text, indicating their inhibitory values in JAK2 and JAK3. Figures were drawn and labeled with ChemDraw v. 19.0.

Sanachai et al. [49] developed a tofacitib-based pharmacophore model to screen 54 pyrazolone derivatives synthesized from an internal data set. From the virtual screening procedure, they selected twelve compounds to test in vitro against both JAKs 2 and 3. In vitro kinase inhibition indicated that compounds **94**, **95**, and **96** (Figure 49) inhibited both JAKs (Figure 49). Compound **95** showed the highest inhibition of protein kinases (Figure 49). The molecular dynamics simulation analysis indicated that the sulfonamide group of compound **95** can form hydrogen bonds with residues Glu930 and Lys932 of JAK2 and Glu903 and Lys905 located in the hinge region of JAK3, but the van der Waals forces also play a role in ligand binding.

**Figure 50:**
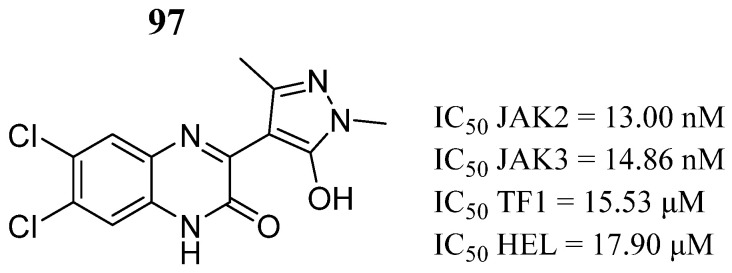
Chemical structure of the lead compound (**97**) identified as a potential quinoxalinone-based dual JAK2/3 inhibitor by Sanachai et al. [105]. In the figure, the number in bold refers to the compound described in the text, indicating their inhibitory values in JAK2 and JAK3, and distinct cancer cell lines. Figures were drawn and labeled with ChemDraw v. 19.0.

In the same year, Sanachai et al. [105] analyzed an internal library of 49 quinoxalinones using a virtual screening based on JAK2 and three proteins. Of them, 17 selected compounds were tested against two human erythroleukemia cell lines, TF1 and HEL. Compound **97** showed strong inhibition against JAK2/3 (Figure 50), with values better than or similar to the reference drugs ruxolitinib and tofacitinib (IC_50_ = 14.50 and 29.09 nM). Furthermore, compound **97** inhibited TF1 cells and HEL cells, similar to the reference drugs (IC_50_ = 29.09 and 23.28 nM, respectively). In addition, compound **97** inhibited JAK2 autophosphorylation and induced cell apoptosis. Molecular dynamics simulations showed that compound **97** was stabilized by van der Waals forces, and the hydroxyl group that forms hydrogen bonds with the Ser936 and Arg938 residues in the hinge region of JAK2.

**Figure 51:**
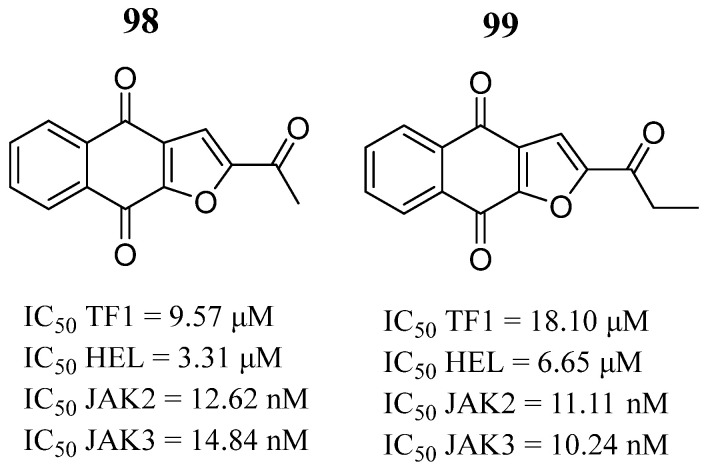
Chemical structures of naphthoquinones identified as potential JAK2/3 inhibitors by Sanachai et al. [106]. In the figure, the numbers in bold refer to the compounds described in the text, indicating their inhibitory values in JAK2 and JAK3, and distinct cancer cell lines. Figures were drawn and labeled with ChemDraw v. 19.0.

Sanachai et al. [106] investigated a series of naphthoquinones to identify new JAK2/3 inhibitors. Napabucasin (**98**) and 2′-methyl napabucasin (**99**) affected the cell growth of the TF1 and HEL erythroleukemia cell lines and significantly inhibited the activity of both kinases (Figure 51) even better than tofacitinib (IC_50_ = 32.10 and 21.03 nM). These two compounds induced apoptosis in TF1 cells in a time- and dose-dependent manner. Both compounds formed hydrogen bonds with residues Tyr931 and Leu932 and had hydrophobic contact with the hinge region, the G-loop, and the catalytic loop of JAK2 along the molecular simulations. Thus, the authors suggested that compounds **98** and **99** are potential candidates for the further development of new anticancer drugs targeting JAKs.

## 9. Summary

The JAK2 and JAK3 protein kinases that participate in the mediation of cytokine signaling are important drug targets for the prevention or regulation of various types of cancers. Our review reveals that the current research focused on synthetic routes devoted to the development of hybrid JAK2/3 inhibitors using preexisting precursors. These newly developed compounds privilege compounds containing heterocyclic aromatic rings such as pyrimidine, pyridine, and pyrazolo [3,4-*d*]pyrimidine. Moreover, inhibitors of natural origin derived from extract plants and insects have shown suitable inhibitory capacities, making it an important area to continue the search for natural compounds. Computer-aided studies indicated that the presence of nonpolar regions and polar substituents, such as nitro and oxo groups, in these structures allows for the variety of interactions observed. The prevalence of interactions with Leu932, Lys882, and Glu930 at the active site of JAK2 suggests that these residues are critical for the design of specific and potent inhibitors. The residue Leu855 is frequently mentioned in hydrophobic interactions and hydrogen bonds. Hydrophobic interactions with Val863 and pi-stacking interactions with Tyr931 are also common. On the other hand, JAK3 presents a different profile with Glu903, Leu905, and Arg953 as the main hydrogen interaction sites. Residue Asp912 also forms hydrogen bonds, although it is less common. On the other hand, Leu828 is present in hydrophobic interactions, and Cys909 is involved in covalent bonds (Table 3). Comparative analysis shows that, although there are similarities in the binding sites of JAK2 and JAK3, key differences in the residues involved can significantly influence the selectivity and efficacy of the inhibitors. Biological evaluation showed that the inhibition of JAK2/3 also impairs the signaling pathway evidenced by the inhibition of distinct STAT signaling molecules. Further preclinical studies are required to obtain full approval of these inhibitors for use in humans. The next step would be to move towards translational medicine to identify which of those JAK2/3 inhibitors (Table 3) can be used for the treatment of cancer.

## Figures and Tables

**Table 1:** JAK2 inhibitor compounds [11,12,13,14,15].

Compound	Activity	Disease	Toxic Effects
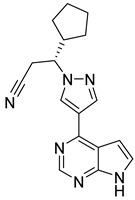 Ruxolitinib	IC_50_ = 2.8 nM	Polycythemia, Myelofibrosis, Various cancers	Diarrhea, abdominal pain, anemia, thrombocytopenia
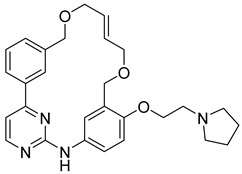 Pacritinib	IC_50_ = 23 nM	Myeloid leukemias, Myelofibrosis	Cardiovascular and hemorrhagic events
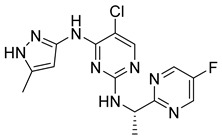 AZD1480	*K_i_* = 0.26 nM	Myeloproliferative diseases, Solid tumors	Dizziness, anxiety, memory loss, ataxia, hallucinations, behavior changes

**Table 2:** JAK3 inhibitor compounds [16,17,18,19,20,21].

Compound	Activity	Disease	Toxic Effects
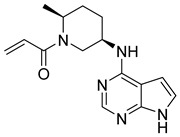 Ritlecitinib	IC_50_ = 33.1 nM	Alopecia areata, Vitiligo, Ulcerative colitis, Rheumatoid arthritis, Crohn’s disease	Hepatotoxicity, Pruritus, Influenza
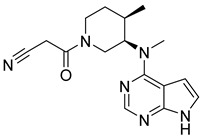 Tofacitinib	IC_50_ = 1 nM	Transplant patients, Autoimmune disease, Rheumatoid arthritis	Infection, Cytopenias

**Table 3:** Summary of potential inhibitors of JAK2 and JAK3.

Compound	Anticancer Activity	Enzyme Activity	In Silico Analysis	References
**JAK2**				
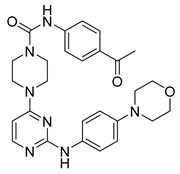 3	GI_50_ HEL = 4.3 μM GI_50_ MV4-11 = 11.0 μM GI_50_ HL60 = 16.5 μM	IC_50_ = 0.027 μM	Binds to the ATP-binding site, two hydrogen bonds with Leu932, a network-like alkyl-π interaction with the adjacent Ala880, Leu855, a hydrogen bond with Lys943 and another with Leu855.	[53]
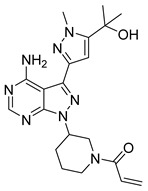 5	IC_50_ HEL = 6.46 μM	IC_50_ = 0.0065 μM	Two hydrogen bonds with Leu932 and two π-π interactions with Tyr931, a hydrogen bond with Lys857, two hydrogen bonds with residue Tyr931, and hydrogen bonding with Ser936 and Asp939 via a water molecule, near the ATP-binding site.	[39]
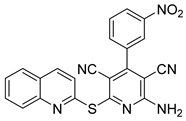 15	IC_50_ MCF-7 = 6.39 µM IC_50_ A549 = 6.9 µM	-	Hydrogen bond with the residue Leu932, lipophilic interactions with the nonpolar amino acid residues Leu83, Leu855, Val863, Pro933, Met929, Ala880 and Leu932 within the receptor pocket.	[70]
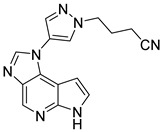 20		IC_50_ = 0.01 μM	Hydrogen bonds with Glu930 and Leu932, hydrogen bonds with Lys882.	[74]
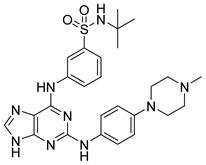 30	-	IC_50_ = 0.022 μM	Hydrogen bonds with Pro375, Lys378, Asp381, Tyr390, and Asn433	[80]
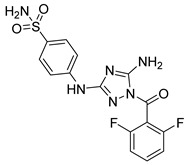 34	IC_50_ HEL = 5.6 µM IC_50_ SET-2 = 5.8 µM	IC_50_ = 0.04 µM	Two hydrogen bonds with Val629 and Glu627, hydrogen bond with the Lys640 side chain of the αD helix.	[28]
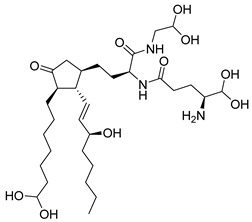 46	-	-	Hydrogen bonding interactions with Lys882, Arg980, Ser936, Asp939, and Lys943, carbon hydrogen bonding interactions with Leu932 and Leu855, and a sulfur bonding interaction with Lue855	[71]
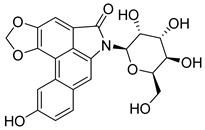 55	-	IC_50_ = 131.8 nM	Interacts with the amino acid residues Ala880, Val863, Tyr931, Leu855 Asp994, Leu983, Asn981 and Arg980	[93]
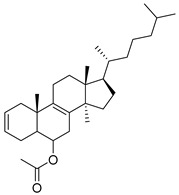 56–67	IC_50_ TNBC = 90.09 to 172.16 μg/mL IC_50_ HEK293T = 732.52 to 1367.25 μg/mL	-	Interaction with residues of catalytic sites such as Leu983, Leu855, Val863, Arg980, Val863, Ala880, Leu855 and Leu932, in the binding pocket	[94]
**JAK3**				
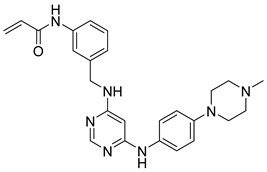 71	-	IC_50_ = 2.1 nM	Bidentate hydrogen bonds with Leu905, and the Cys909, van der Waals contact with Leu956 and Leu828 in the ATP-binding pocket, a hydrogen bond with Arg953	[96]
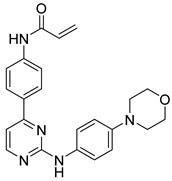 72	-	IC_50_ = 1.7 nM	Bidentate hinge hydrogen bonds with Leu905, covalent bonds with Cys909, two σ-π interactions and one σ-π interaction with amino acid residues Leu828 and Gly908, a hydrogen bond with Asp912	[20]
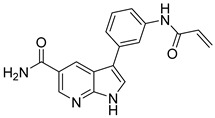 73	-	IC_50_ < 0.1 nM	Hinge interaction pattern and the covalent binding of Cys909, two hydrogen bonds with Lys905, a hydrogen bond with Glu903.	[17]
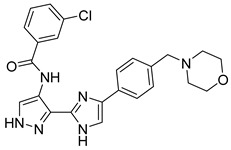 74	IC_50_ K562 = 6.72 µM	IC_50_ = 0.057 µM	Interaction at the binding site with Glu930, Tyr931, Leu932, Ser936 and Gly993.	[97]
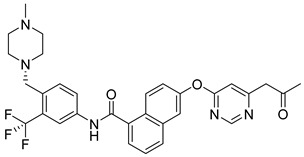 81	IC_50_ MOLM-16 = 1.83 nM	IC_50_ = 4.11 nM	Hydrogen bond interaction with Lys855, hydrogen bond interaction with Leu905, hydrophobic interactions with Leu905, Gly906, and Arg953	[99]
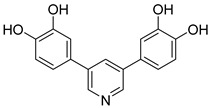 92	IC_50_ A549 = 1.68 µM IC_50_ Huh-7 = 4.88 µM IC_50_ K562 = 2.13 µM	IC_50_ = 1.72 µM	-	[103]
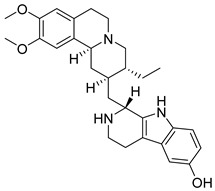 93	-	EC_50_ = 1.4 μmol/L	Interaction with Val812, Ala829, Glu847, Met878, Leu881, Leu932 y Asp943.	[104]
JAK2/3				
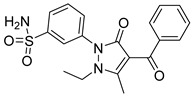 95	-	IC_50_ JAK2 = 12.61 nM IC_50_ JAK3 = 15.80 nM	Hydrogen bonds in the hinge region with residues Glu930 and Lys932 of JAK2 and Glu903 and Lys905.	[49]
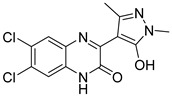 97	IC_50_ TF1 = 15.53 μM IC_50_ HEL = 17.90 μM	IC_50_ JAK2 = 13.00 nM IC_50_ JAK3 = 14.86 nM	Hydrogen bonds in the hinge region with residues Ser936 and Arg938.	[105]
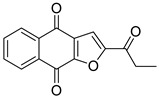 99	IC_50_ TF1 = 18.10 μM IC_50_ HEL = 6.65 μM	IC_50_ JAK2 = 11.11 nM IC_50_ JAK3 = 10.24 nM	Hydrogen bonds with residues Y931 and L932 and hydrophobic contact with the hinge region, the G loop and the catalytic loop.	[106]

## References

[1] Bose S., Banerjee S., Mondal A., Chakraborty U., Pumarol J., Croley C.R., Bishayee A. (2020). Targeting the JAK/STAT Signaling Pathway Using Phytocompounds for Cancer Prevention and Therapy. Cells.

[2] Jemal A., Siegel R., Xu J., Ward E. (2010). Cancer statistics. CA Cancer J. Clin..

[3] Leonard W.J., O’Shea J.J. (1998). Jaks and STATs: Biological implications. Annu. Rev. Immunol..

[4] Ihle J.N., Witthuhn B.A., Quelle F.W., Yamamoto K., Silvennoinen O. (1995). Signaling through the hematopoietic cytokine receptors. Annu. Rev. Immunol..

[5] Hammarén H.M., Virtanen A.T., Raivola J., Silvennoinen O. (2019). The regulation of JAKs in cytokine signaling and its breakdown in disease. Cytokine.

[6] Haan C., Kreis S., Margue C., Behrmann I. (2006). Jaks and cytokine receptors–an intimate relationship. Biochem. Pharmacol..

[7] Pölläniemi A., Virtanen A., Silvennoinen O., Haikarainen T. (2023). Development of an enzyme-coupled activity assay for Janus kinase 2 inhibitor screening. SLAS Discov..

[8] O’Shea J.J., Schwartz D.M., Villarino A.V., Gadina M., McInnes I.B., Laurence A. (2015). The JAK-STAT pathway: Impact on human disease and therapeutic intervention. Annu. Rev. Med..

[9] Schwartz D.M., Kanno Y., Villarino A., Ward M., Gadina M., O’Shea J.J. (2017). JAK inhibition as a therapeutic strategy for immune and inflammatory diseases. Nat. Rev. Drug Discov..

[10] Li Y., Guo F., Chen T., Zhang L., Qin Y. (2021). Anthraquinone derivative C10 inhibits proliferation and cell cycle progression in colon cancer cells via the Jak2/Stat3 signaling pathway. Toxicol. Appl. Pharmacol..

[11] Pardanani A., Gotlib J.R., Jamieson C., Cortes J.E., Talpaz M., Stone R.M., Silverman M.H., Gilliland D.G., Shorr J., Tefferi A. (2011). Safety and efficacy of TG101348, a selective JAK2 inhibitor, in myelofibrosis. J. Clin. Oncol..

[12] Harrison C., Kiladjian J.J., Al-Ali H.K., Gisslinger H., Waltzman R., Stalbovskaya V., McQuitty M., Hunter D.S., Levy R., Knoops L. (2012). JAK inhibition with ruxolitinib versus best available therapy for myelofibrosis. N. Engl. J. Med..

[13] Plimack E.R., Lorusso P.M., McCoon P., Tang W., Krebs A.D., Curt G., Eckhardt S.G. (2013). AZD1480: A phase I study of a novel JAK2 inhibitor in solid tumors. Oncol..

[14] Quintás-Cardama A., Vaddi K., Liu P., Manshouri T., Li J., Scherle P.A., Caulder E., Wen X., Li Y., Waeltz P. (2010). Preclinical characterization of the selective JAK1/2 inhibitor INCB018424: Therapeutic implications for the treatment of myeloproliferative neoplasms. Blood.

[15] Bose P., Verstovsek S. (2017). JAK2 inhibitors for myeloproliferative neoplasms: What is next?. Blood.

[16] Telliez J.B., Dowty M.E., Wang L., Jussif J., Lin T., Li L., Moy E., Balbo P., Li W., Zhao Y. (2016). Discovery of a JAK3-selective inhibitor: Functional differentiation of JAK3-selective inhibition over pan-JAK or JAK1-selective inhibition. ACS Chem. Biol..

[17] Forster M., Liang X.J., Schröder M., Gerstenecker S., Chaikuad A., Knapp S., Laufer S., Gehringer M. (2020). Discovery of a Novel Class of Covalent Dual Inhibitors Targeting the Protein Kinases BMX and BTK. Int. J. Mol. Sci..

[18] Ramírez-Marín H.A., Tosti A. (2022). Evaluating the Therapeutic Potential of Ritlecitinib for the Treatment of Alopecia Areata. Drug Des. Dev. Ther..

[19] Jiang J.K., Ghoreschi K., Deflorian F., Chen Z., Perreira M., Pesu M., Smith J., Nguyen D.T., Liu E.H., Leister W. (2008). Examining the chirality, conformation and selective kinase inhibition of 3-((3R,4R)-4-methyl-3-(methyl(7H-pyrrolo[2,3-d]pyrimidin-4-yl)amino)piperidin-1-yl)-3-oxopropanenitrile (CP-690,550). J. Med. Chem..

[20] Shu L., Chen C., Huan X., Huang H., Wang M., Zhang J., Yan Y., Liu J., Zhang T., Zhang D. (2020). Design, synthesis, and pharmacological evaluation of 4- or 6-phenyl-pyrimidine derivatives as novel and selective Janus kinase 3 inhibitors. Eur. J. Med. Chem..

[21] Su W., Chen Z., Liu M., He R., Liu C., Li R., Gao M., Zheng M., Tu Z., Zhang Z. (2022). Design, synthesis and structure-activity relationship studies of pyrido[2,3-d]pyrimidin-7-ones as potent Janus Kinase 3 (JAK3) covalent inhibitors. Bioorg. Med. Chem. Lett..

[22] Wang W., Diao Y., Li W., Luo Y., Yang T., Zhao Y., Qi T., Xu F., Ma X., Ge H. (2019). Design, synthesis and structure-activity relationship study of aminopyridine derivatives as novel inhibitors of Janus kinase 2. Bioorg. Med. Chem. Lett..

[23] Cook A.M., Li L., Ho Y., Lin A., Li L., Stein A., Forman S., Perrotti D., Jove R., Bhatia R. (2014). Role of altered growth factor receptor-mediated JAK2 signaling in growth and maintenance of human acute myeloid leukemia stem cells. Blood.

[24] Dosil M., Wang S., Lemischka I.R. (1993). Mitogenic signalling and substrate specificity of the Flk2/Flt3 receptor tyrosine kinase in fibroblasts and interleukin 3-dependent hematopoietic cells. Mol. Cell. Biol..

[25] Wu P., Nielsen T.E., Clausen M.H. (2015). FDA-approved small-molecule kinase inhibitors. Trends Pharmacol. Sci..

[26] Alicea-Velázquez N.L., Boggon T.J. (2011). The use of structural biology in Janus kinase targeted drug discovery. Curr. Drug Targets.

[27] Ghoreschi K., Laurence A., O’Shea J.J. (2009). Janus kinases in immune cell signaling. Immunol. Rev..

[28] Virtanen A.T., Haikarainen T., Sampathkumar P., Palmroth M., Liukkonen S., Liu J., Nekhotiaeva N., Hubbard S.R., Silvennoinen O. (2023). Identification of Novel Small Molecule Ligands for JAK2 Pseudokinase Domain. Pharmaceuticals.

[29] Loh C.Y., Arya A., Naema A.F., Wong W.F., Sethi G., Looi C.Y. (2019). Signal Transducer and Activator of Transcription (STATs) Proteins in Cancer and Inflammation: Functions and Therapeutic Implication. Front. Oncol..

[30] Doheny D., Sirkisoon S., Carpenter R.L., Aguayo N.R., Regua A.T., Anguelov M., Manore S.G., Arrigo A., Jalboush S.A., Wong G.L. (2020). Combined inhibition of JAK2-STAT3 and SMO-GLI1/tGLI1 pathways suppresses breast cancer stem cells, tumor growth, and metastasis. Oncogene.

[31] Johnson D.E., O’Keefe R.A., Grandis J.R. (2018). Targeting the IL-6/JAK/STAT3 signalling axis in cancer. Nat. Rev. Clin. Oncol..

[32] Wang Y., Shen Y., Wang S., Shen Q., Zhou X. (2018). The role of STAT3 in leading the crosstalk between human cancers and the immune system. Cancer Lett..

[33] Raivola J., Haikarainen T., Silvennoinen O. (2020). Characterization of JAK1 Pseudokinase Domain in Cytokine Signaling. Cancers.

[34] Raivola J., Hammarén H.M., Virtanen A.T., Bulleeraz V., Ward A.C., Silvennoinen O. (2018). Hyperactivation of Oncogenic JAK3 Mutants Depend on ATP Binding to the Pseudokinase Domain. Front. Oncol..

[35] James C., Ugo V., Le Couédic J.P., Staerk J., Delhommeau F., Lacout C., Garçon L., Raslova H., Berger R., Bennaceur-Griscelli A. (2005). A unique clonal JAK2 mutation leading to constitutive signalling causes polycythaemia vera. Nature.

[36] Baxter E.J., Scott L.M., Campbell P.J., East C., Fourouclas N., Swanton S., Vassiliou G.S., Bench A.J., Boyd E.M., Curtin N. (2005). Acquired mutation of the tyrosine kinase JAK2 in human myeloproliferative disorders. Lancet.

[37] Kralovics R., Passamonti F., Buser A.S., Teo S.S., Tiedt R., Passweg J.R., Tichelli A., Cazzola M., Skoda R.C. (2005). A gain-of-function mutation of JAK2 in myeloproliferative disorders. N. Engl. J. Med..

[38] Zhao R., Xing S., Li Z., Fu X., Li Q., Krantz S.B., Zhao Z.J. (2005). Identification of an acquired JAK2 mutation in polycythemia vera. J. Biol. Chem..

[39] Yin Y., Chen C.J., Yu R.N., Shu L., Zhang T.T., Zhang D.Y. (2019). Discovery of novel selective Janus kinase 2 (JAK2) inhibitors bearing a 1H-pyrazolo[3,4-d]pyrimidin-4-amino scaffold. Bioorg. Chem..

[40] Xiong H., Du W., Zhang Y.J., Hong J., Su W.Y., Tang J.T., Wang Y.C., Lu R., Fang J.Y. (2012). Trichostatin A, a histone deacetylase inhibitor, suppresses JAK2/STAT3 signaling via inducing the promoter-associated histone acetylation of SOCS1 and SOCS3 in human colorectal cancer cells. Mol. Carcinog..

[41] Zhang X., Hu F., Li G., Li G., Yang X., Liu L., Zhang R., Zhang B., Feng Y. (2018). Human colorectal cancer-derived mesenchymal stem cells promote colorectal cancer progression through IL-6/JAK2/STAT3 signaling. Cell Death Dis..

[42] Leroy E., Constantinescu S.N. (2017). Rethinking JAK2 inhibition: Towards novel strategies of more specific and versatile janus kinase inhibition. Leukemia.

[43] Zhang J., Yang P.L., Gray N.S. (2009). Targeting cancer with small molecule kinase inhibitors. Nat. Rev. Cancer.

[44] O’Shea J.J., Gadina M., Schreiber R.D. (2002). Cytokine signaling in 2002: New surprises in the Jak/Stat pathway. Cell.

[45] Ren J., Shi W., Zhao D., Wang Q., Chang X., He X., Wang X., Gao Y., Lu P., Zhang X. (2020). Design and synthesis of boron-containing diphenylpyrimidines as potent BTK and JAK3 dual inhibitors. Bioorg. Med. Chem..

[46] Sanachai K., Mahalapbutr P., Hengphasatporn K., Shigeta Y., Seetaha S., Tabtimmai L., Langer T., Wolschann P., Kittikool T., Yotphan S. (2022). Pharmacophore-Based Virtual Screening and Experimental Validation of Pyrazolone-Derived Inhibitors toward Janus Kinases. ACS Omega.

[47] Lai R., Rassidakis G.Z., Lin Q., Atwell C., Medeiros L.J., Amin H.M. (2005). Jak3 activation is significantly associated with ALK expression in anaplastic large cell lymphoma. Hum. Pathol..

[48] Gee K., Kozlowski M., Kryworuchko M., Diaz-Mitoma F., Kumar A. (2001). Differential effect of IL-4 and IL-13 on CD44 expression in the Burkitt’s lymphoma B cell line BL30/B95-8 and in Epstein-Barr virus (EBV) transformed human B cells: Loss of IL-13 receptors on Burkitt’s lymphoma B cells. Cell. Immunol..

[49] Yared M.A., Khoury J.D., Medeiros L.J., Rassidakis G.Z., Lai R. (2005). Activation status of the JAK/STAT3 pathway in mantle cell lymphoma. Arch. Pathol. Lab. Med..

[50] Malamut G., El Machhour R., Montcuquet N., Martin-Lannerée S., Dusanter-Fourt I., Verkarre V., Mention J.J., Rahmi G., Kiyono H., Butz E.A. (2010). IL-15 triggers an antiapoptotic pathway in human intraepithelial lymphocytes that is a potential new target in celiac disease-associated inflammation and lymphomagenesis. J. Clin. Investig..

[51] Forster M., Gehringer M., Laufer S.A. (2017). Recent advances in JAK3 inhibition: Isoform selectivity by covalent cysteine targeting. Bioorg. Med. Chem. Lett..

[52] Mohamed S.A., El-Kady D.S., Abd-Rabou A.A., Tantawy M.A., AbdElhalim M.M., Elazabawy S.R., Abdallah A.E.M., Elmegeed G.A. (2020). Synthesis of novel hybrid hetero-steroids: Molecular docking study augmented anti-proliferative properties against cancerous cells. Steroids.

[53] Li Y., Ye T., Xu L., Dong Y., Luo Y., Wang C., Han Y., Chen K., Qin M., Liu Y. (2019). Discovery of 4-piperazinyl-2-aminopyrimidine derivatives as dual inhibitors of JAK2 and FLT3. Eur. J. Med. Chem..

[54] Jyothi-Buggana S., Paturi M.C., Perka H., Gade D.R., Vvs R.P. (2019). Novel 2,4-disubstituted quinazolines as cytotoxic agents and JAK2 inhibitors: Synthesis, in vitro evaluation and molecular dynamics studies. Comput. Biol. Chem..

[55] Sinha I., Null K., Wolter W., Suckow M.A., King T., Pinto J.T., Sinha R. (2012). Methylseleninic acid downregulates hypoxia-inducible factor-1α in invasive prostate cancer. Int. J. Cancer.

[56] Tarrado-Castellarnau M., Cortés R., Zanuy M., Tarragó-Celada J., Polat I.H., Hill R., Fan T.W., Link W., Cascante M. (2015). Methylseleninic acid promotes antitumour effects via nuclear FOXO3a translocation through Akt inhibition. Pharmacol. Res..

[57] Zhang T., Zhu X., Qiu J., Jiang K., Zhao G., Wu H., Deng G., Qiu C. (2021). Correction to: Methylseleninic Acid Suppresses Breast Cancer Growth via the JAK2/STAT3 Pathway. Reprod. Sci..

[58] Ma X., Diao Y., Ge H., Xu F., Zhu L., Zhao Z., Li H. (2020). Discovery and optimization of 2-aminopyridine derivatives as novel and selective JAK2 inhibitors. Bioorg. Med. Chem. Lett..

[59] Li Y., Wang P., Chen C., Ye T., Han Y., Hou Y., Liu Y., Gong P., Qin M., Zhao Y. (2020). Discovery and rational design of 2-aminopyrimidine-based derivatives targeting Janus kinase 2 (JAK2) and FMS-like tyrosine kinase 3 (FLT3). Bioorg. Chem..

[60] Bottiglieri T. (2002). S-Adenosyl-L-methionine (SAMe): From the bench to the bedside—Molecular basis of a pleiotrophic molecule. Am. J. Clin. Nutr..

[61] Mahmood N., Cheishvili D., Arakelian A., Tanvir I., Khan H.A., Pépin A.S., Szyf M., Rabbani S.A. (2017). Methyl donor S-adenosylmethionine (SAM) supplementation attenuates breast cancer growth, invasion, and metastasis in vivo; therapeutic and chemopreventive applications. Oncotarget.

[62] Ma D., Shen B., Seewoo V., Tong H., Yang W., Cheng X., Jin Z., Peng C., Qiu W. (2016). GADD45β induction by S-adenosylmethionine inhibits hepatocellular carcinoma cell proliferation during acute ischemia-hypoxia. Oncotarget.

[63] Parashar S., Cheishvili D., Arakelian A., Hussain Z., Tanvir I., Khan H.A., Szyf M., Rabbani S.A. (2015). S-adenosylmethionine blocks osteosarcoma cells proliferation and invasion in vitro and tumor metastasis in vivo: Therapeutic and diagnostic clinical applications. Cancer Med..

[64] Li T.W., Zhang Q., Oh P., Xia M., Chen H., Bemanian S., Lastra N., Circ M., Moyer M.P., Mato J.M. (2009). S-Adenosylmethionine and methylthioadenosine inhibit cellular FLICE inhibitory protein expression and induce apoptosis in colon cancer cells. Mol. Pharmacol..

[65] Liu Y., Bi T., Yuan F., Gao X., Jia G., Tian Z. (2020). S-adenosylmethionine induces apoptosis and cycle arrest of gallbladder carcinoma cells by suppression of JAK2/STAT3 pathways. Naunyn-Schmiedeberg’s Arch. Pharmacol..

[66] Speich B., Ame S.M., Ali S.M., Alles R., Hattendorf J., Utzinger J., Albonico M., Keiser J. (2012). Efficacy and safety of nitazoxanide, albendazole, and nitazoxanide-albendazole against Trichuris trichiura infection: A randomized controlled trial. PLoS Negl. Trop. Dis..

[67] Stockis A., Allemon A.M., De Bruyn S., Gengler C. (2002). Nitazoxanide pharmacokinetics and tolerability in man using single ascending oral doses. Int. J. Clin. Pharmacol. Ther..

[68] Di Santo N., Ehrisman J. (2013). Research perspective: Potential role of nitazoxanide in ovarian cancer treatment. Old drug, new purpose?. Cancers.

[69] Tantawy M.A., El-Sherbeeny N.A., Helmi N., Alazragi R., Salem N., Elaidy S.M. (2020). Synthetic antiprotozoal thiazolide drug induced apoptosis in colorectal cancer cells: Implications of IL-6/JAK2/STAT3 and p53/caspases-dependent signaling pathways based on molecular docking and in vitro study. Mol. Cell Biochem..

[70] Nafie M.S., Mahgoub S., Amer A.M. (2021). Antimicrobial and antiproliferative activities of novel synthesized 6-(quinolin-2-ylthio) pyridine derivatives with molecular docking study as multi-targeted JAK2/STAT3 inhibitors. Chem. Biol. Drug Des..

[71] Li W., Yuan B., Zhao Y., Lu T., Zhang S., Ding Z., Wang D., Zhong S., Gao G., Yan M. (2021). Transcriptome profiling reveals target in primary myelofibrosis together with structural biology study on novel natural inhibitors regarding JAK2. Aging.

[72] Xie R.F., Li Z.C., Chen P.P., Zhou X. (2015). Bufothionine induced the mitochondria-mediated apoptosis in H22 liver tumor and acute liver injury. Chin. Med..

[73] Kong W.S., Shen F.X., Xie R.F., Zhou G., Feng Y.M., Zhou X. (2021). Bufothionine induces autophagy in H22 hepatoma-bearing mice by inhibiting JAK2/STAT3 pathway, a possible anti-cancer mechanism of cinobufacini. J. Ethnopharmacol..

[74] Xu P., Shen P., Wang H., Qin L., Ren J., Sun Q., Ge R., Bian J., Zhong Y., Li Z. (2021). Discovery of imidazopyrrolopyridines derivatives as novel and selective inhibitors of JAK2. Eur. J. Med. Chem..

[75] Sanachai K., Aiebchun T., Mahalapbutr P., Seetaha S., Tabtimmai L., Maitarad P., Xenikakis I., Geronikaki A., Choowongkomon K., Rungrotmongkol T. (2021). Discovery of novel JAK2 and EGFR inhibitors from a series of thiazole-based chalcone derivatives. RSC Med. Chem..

[76] Newton A.S., Liosi M.E., Henry S.P., Deiana L., Faver J.C., Krimmer S.G., Puleo D.E., Schlessinger J., Jorgensen W.L. (2021). Indoloxytriazines as binding molecules for the JAK2 JH2 pseudokinase domain and its V617F variant. Tetrahedron Lett..

[77] Tantawy M.A., Shaheen S., Kattan S.W., Alelwani W., Barnawi I.O., Elmgeed G.A., Nafie M.S. (2022). Cytotoxicity, in silico predictions and molecular studies for androstane heterocycle compounds revealed potential antitumor agent against lung cancer cells. J. Biomol. Struct. Dyn..

[78] Singh A., Mishra A. (2023). Molecular modelling study to discover novel JAK2 signaling pathway inhibitor. J. Biomol. Struct. Dyn..

[79] He L., Liu J., Zhao H.L., Zhang L.C., Yu R.L., Kang C.M. (2023). De novo design of dual-target JAK2, SMO inhibitors based on deep reinforcement learning, molecular docking and molecular dynamics simulations. Biochem. Biophys. Res. Commun..

[80] Guo Y., Zou Y., Chen Y., Deng D., Zhang Z., Liu K., Tang M., Yang T., Fu S., Zhang C. (2023). Design, synthesis and biological evaluation of purine-based derivatives as novel JAK2/BRD4(BD2) dual target inhibitors. Bioorg. Chem..

[81] Diao Y., Liu D., Ge H., Zhang R., Jiang K., Bao R., Zhu X., Bi H., Liao W., Chen Z. (2023). Macrocyclization of linear molecules by deep learning to facilitate macrocyclic drug candidates discovery. Nat. Commun..

[82] Suriya U., Mahalapbutr P., Geronikaki A., Kartsev V., Zubenko A., Divaeva L., Chekrisheva V., Petrou A., Oopkaew L., Somngam P. (2024). Discovery of furopyridine-based compounds as novel inhibitors of Janus kinase 2: In silico and in vitro studies. Int. J. Biol. Macromol..

[83] Yasir M., Park J., Han E.T., Park W.S., Han J.H., Chun W. (2024). Drug Repositioning via Graph Neural Networks: Identifying Novel JAK2 Inhibitors from FDA-Approved Drugs through Molecular Docking and Biological Validation. Molecules.

[84] Kubo I., Muroi H., Himejima M. (1993). Combination effects of antifungal nagilactones against *Candida albicans* and two other fungi with phenylpropanoids. J. Nat. Prod..

[85] Shan H., Yao S., Ye Y., Yu Q. (2019). 3-Deoxy-2β,16-dihydroxynagilactone E, a natural compound from *Podocarpus nagi*, preferentially inhibits JAK2/STAT3 signaling by allosterically interacting with the regulatory domain of JAK2 and induces apoptosis of cancer cells. Acta Pharmacol. Sin..

[86] Huang H., Niu J., Wang F., Hu L., Yu Q. (2019). A natural compound derivative P-13 inhibits STAT3 signaling by covalently inhibiting Janus kinase 2. Investig. New Drugs.

[87] Jiang D., Xu J., Liu S., Nasser M.I., Wei W., Mao T., Liu X., Zou X., Li J., Li X. (2021). Rosmanol induces breast cancer cells apoptosis by regulating PI3K/AKT and STAT3/JAK2 signaling pathways. Oncol. Lett..

[88] Zhong Y.Y., Chen H.P., Tan B.Z., Yu H.H., Huang X.S. (2013). Triptolide avoids cisplatin resistance and induces apoptosis via the reactive oxygen species/nuclear factor-κB pathway in SKOV3PT platinum-resistant human ovarian cancer cells. Oncol. Lett..

[89] Wei Y.M., Wang Y.H., Xue H.Q., Luan Z.H., Liu B.W., Ren J.H. (2019). Triptolide, A potential autophagy modulator. Chin. J. Integr. Med..

[90] Zhong Y., Le F., Cheng J., Luo C., Zhang X., Wu X., Xu F., Zuo Q., Tan B. (2021). Triptolide inhibits JAK2/STAT3 signaling and induces lethal autophagy through ROS generation in cisplatin-resistant SKOV3/DDP ovarian cancer cells. Oncol. Rep..

[91] Wu Y., Liu C., Niu Y., Xia J., Fan L., Wu Y., Gao W. (2021). Procyanidins mediates antineoplastic effects against non-small cell lung cancer via the JAK2/STAT3 pathway. Transl. Cancer Res..

[92] Park K.H., Joo S.H., Seo J.H., Kim J., Yoon G., Jeon Y.J., Lee M.H., Chae J.I., Kim W.K., Shim J.H. (2022). Licochalcone H Induces Cell Cycle Arrest and Apoptosis in Human Skin Cancer Cells by Modulating JAK2/STAT3 Signaling. Biomol. Ther..

[93] Shaikh A. (2023). Computational modeling and in vitro evaluation identified natural product-Z218 as a novel Janus kinase 2 (JAK2) inhibitor to combat β-thalassemia. Biotechnol. Appl. Biochem..

[94] Upreti S., Muduli K., Pradhan J., Elangovan S., Samant M. (2023). Identification of novel inhibitors from *Urtica* spp. against TNBC targeting JAK2 receptor for breast cancer therapy. Med. Oncol..

[95] Vaziri-Amjad S., Rahgosha R., Taherkhani A. (2024). Potential JAK2 Inhibitors from Selected Natural Compounds: A Promising Approach for Complementary Therapy in Cancer Patients. Evid. Based Complement. Alternat. Med..

[96] Yu R.N., Chen C.J., Shu L., Yin Y., Wang Z.J., Zhang T.T., Zhang D.Y. (2019). Structure-based design and synthesis of pyrimidine-4,6-diamine derivatives as Janus kinase 3 inhibitors. Bioorg. Med. Chem..

[97] Zheng Y.G., Wang J.A., Meng L., Pei X., Zhang L., An L., Li C.L., Miao Y.L. (2021). Design, synthesis, biological activity evaluation of 3-(4-phenyl-1H-imidazol-2-yl)-1H-pyrazole derivatives as potent JAK 2/3 and aurora A/B kinases multi-targeted inhibitors. Eur. J. Med. Chem..

[98] Medvedeva S.M., Shikhaliev K.S. (2022). Synthesis of 4,5-Dihydro-1H-[1,2]dithiolo[3,4-c]quinoline-1-thione Derivatives and Their Application as Protein Kinase Inhibitors. Molecules.

[99] Wei J., Pan Y., Shen Z., Shen L., Xu L., Yu W., Huang W. (2023). A hybrid energy-based and AI-based screening approach for the discovery of novel inhibitors of JAK3. Front. Med..

[100] Faris A., Cacciatore I., Ibrahim I.M., Al-Mughram M.H., Hadni H., Tabti K., Elhallaoui M. (2023). In silico computational drug discovery: A Monte Carlo approach for developing a novel JAK3 inhibitors. J. Biomol. Struct. Dyn..

[101] Faris A., Ibrahim I.M., Alnajjar R., Hadni H., Bhat M.A., Yaseen M., Chakraborty S., Alsakhen N., Shamkh I.M., Mabood F.M. (2023). QSAR-driven screening uncovers and designs novel pyrimidine-4,6-diamine derivatives as potent JAK3 inhibitors. J. Biomol. Struct. Dyn..

[102] Su D., Gao Y.Q., Deng Y.J., Zhang H.H., Wu Y.R., Hu Y., Mei Q.X. (2019). Identification of Chinese Herbal Compounds with Potential as JAK3 Inhibitors. Evid. Based Complement. Alternat. Med..

[103] Yan Y.M., Xu T., Tu Z.C., Zhu H.J., Cheng Y.X. (2020). Sulfur and nitrogen-containing compounds from the whole bodies of *Blaps japanensis*. Bioorg. Chem..

[104] Kim B.H., Yi E.H., Jee J.G., Jeong A.J., Sandoval C., Park I.C., Baeg G.H., Ye S.K. (2020). Tubulosine selectively inhibits JAK3 signalling by binding to the ATP-binding site of the kinase of JAK3. J. Cell Mol. Med..

[105] Sanachai K., Mahalapbutr P., Tabtimmai L., Seetaha S., Kittikool T., Yotphan S., Choowongkomon K., Rungrotmongkol T. (2022). Discovery of JAK2/3 Inhibitors from Quinoxalinone-Containing Compounds. ACS Omega.

[106] Sanachai K., Mahalapbutr P., Tabtimmai L., Seetaha S., Kaekratoke N., Chamni S., Azam S.S., Choowongkomon K., Rungrotmongkol T. (2023). In Silico and In Vitro Study of Janus Kinases Inhibitors from Naphthoquinones. Molecules.

